# Invasive *Lupinus polyphyllus* Alters Functional Traits and Life Strategies of Native Species

**DOI:** 10.1002/ece3.73911

**Published:** 2026-06-29

**Authors:** Marta Czarniecka‐Wiera, Boglárka Berki, Tomasz H. Szymura, Magdalena Szymura

**Affiliations:** ^1^ Institute of Agroecology and Plant Production Wrocław University of Environmental and Life Sciences Wrocław Poland; ^2^ Doctoral School of Biology, Institute of Biology Eötvös Loránd University Budapest Hungary; ^3^ National Laboratory for Health Security, Institute of Ecology and Botany Centre for Ecological Research Vácrátót Hungary; ^4^ Botanical Garden University of Wrocław Wrocław Poland

**Keywords:** CSR strategy, environmental factors, impact, *Lupinus poluphyllus*, native plants, plant functional traits

## Abstract

The primary trait of invasive plant species is their enhanced competitive ability compared to native flora. Consequently, in ecosystems they invade, native species diversity often declines. However, some native plants may allocate resources to developing traits that support their coexistence with invasive species. We conducted an observational experiment to assess how the functional traits of native plants are influenced by the invasion of *Lupinus polyphyllus,* how the Competition‐Stress‐Ruderal (CSR) strategies of native species shift during this process, as well as whether environmental factors modify these relations. We selected eight species (four herbs and four grasses) commonly found in the grasslands of the Sudety Mountains (Poland). Plants and soil were sampled from grasslands dominated by *L. polyphyllus* (> 50% cover) and from adjacent patches free of invasive plants. We measured plant height, leaf area (LA), specific leaf area (SLA), and leaf dry matter content (LDMC). Subsequently, we evaluated each species' allocation to a particular life strategy using the CSR framework. Our findings show that native grassland species growing alongside *L. polyphyllus* tend to be taller, have larger leaves, exhibit higher SLA, and have lower LDMC compared to those in noninvaded areas. Simultaneously, *L. polyphyllus* presence is associated with reduced impacts of K and moisture on herbs' functional traits; C, N, C/N, and heat exposure on grasses' functional traits; as well as low pH and clay content on both groups' functional traits. These shifts in leaf traits among plants interacting with the invader suggest a reduced investment in stress‐tolerance strategies, accompanied by increased investment in competitive or ruderal strategies.

## Graphical abstract

In grasslands invaded by *Lupinus polyphyllus* , native plant species exhibit shifts in functional traits toward increased height, larger leaf area, higher specific leaf area, and lower leaf dry matter content compared to noninvaded sites. These changes indicate a reduced investment in stress tolerance and a greater allocation to competitive or ruderal strategies. At the same time, the invader buffers the influence of certain environmental stressors on native plants.

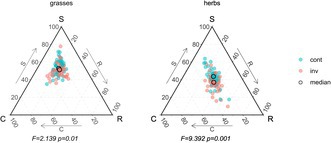

## 1 Introduction

The main characteristic of invasive plant species is their enhanced competitive ability relative to native plants (Vilà and Weiner 2004; Van Kleunen et al. 2010). As a result, in communities invaded by such plants, native species richness and diversity often decline due to competitive exclusion (Hejda et al. 2009; Czarniecka‐Wiera et al. 2019). Nevertheless, some native plants are capable of coexisting with invaders (Strauss et al. 2006; Leger and Espeland 2010). Growing evidence suggests that native species can adapt in response to competitive pressure from invaders (Dostál 2022). For instance, Leger (2008) demonstrated in an experiment with the native perennial grass *Elymus multisetus* M.E. Jones, exposed to the invasion of cheatgrass ( *Bromus tectorum* L.), that plants from invaded sites responded more rapidly to watering and produced more leaves compared to individuals from uninvaded sites. These findings suggest that native species in contact with invaders may invest in traits that enhance competitiveness. Potential mechanisms enabling them to adapt to invasions include phenotypic plasticity, defined as the ability of organisms to modify their morphology and/or physiology in response to changing environmental conditions (Oduor 2013). It has been repeatedly suggested that many native plants exhibit sufficient phenotypic plasticity, which may allow them to adapt to novel habitat conditions created by invasive species (Leger and Espeland 2010; Godoy et al. 2011; Dostál 2022).

The phenotypic plasticity of functional (adaptive) plant traits, which reflect plant resource economics (Sultan 2000), allows for comparing organismal function within and across ecosystems by analyzing changes in functional traits (Van Kleunen et al. 2010; Felker‐Quinn et al. 2013; Díaz et al. 2016). A practical framework for such comparisons is Grimes's CSR strategy scheme (Grime and Pierce 2012), which is widely used to investigate and interpret various community processes, including resistance and resilience, coexistence, succession, and the relationship between species richness and productivity (Lepš et al. 1982; Caccianiga et al. 2006; Cerabolini et al. 2016). A plant's investment in stress tolerance, ruderal behavior, or competitive ability can be assessed using basic functional traits such as leaf area (LA), leaf dry matter content (LDMC), and specific leaf area (SLA) (Pierce et al. 2017). These traits are part of the leaf economics spectrum, a component of the broader “fast–slow” plant economics continuum (Reich 2014), with leaf size variation being a key element of CSR strategy differentiation (Pierce et al. 2012). The relative investment does not represent leaf economics and/or plant size per se, but is the trade‐off between these multiple functions. Particularly, the decreasing LDMC and increasing SLA represent the more acquisitive resource economic spectrum (from S to R strategy), while the increasing LA represents the growing investment into C selection (Pierce et al. 2013, 2022; Díaz et al. 2016). Ecologically, the leaf economics spectrum is applied to evaluate changes in CSR strategies in response to environmental factors, including plant invasion (Lepš et al. 1982; Hunt et al. 2004; Hansen et al. 2021). While most studies on plant traits in the context of biological invasions have focused on shifts in invasive species traits in their new range or compared invasive traits with those of natives (Van Kleunen et al. 2010; Felker‐Quinn et al. 2013; Rotter and Holeski 2018; Gruntman and Segev 2024), much less is known about how native species' functional traits change within invaded communities (Sheppard and Brendel 2021).

*L. polyphyllus* Lindl. is one of Europe's most widespread alien invasive plant species. Originally introduced from North America for ornamental purposes and soil improvement, it has spread extensively and become dominant in various ecosystems. It is considered invasive in several European countries, including Norway, Lithuania, Latvia, and Germany, as well as in regions beyond Europe, such as New Zealand (Fremstad 2010). In Germany, *L. polyphyllus* is among the 15 most common invasive plants and is listed on the national blacklist of invasive species (Nehring et al. 2013). In invaded plant communities, this species can outcompete smaller plants for light due to its tall growth form, thereby reducing species richness (Otte and Maul 2005; Thiele et al. 2010; Ramula and Pihlaja 2012; Hansen et al. 2021). This competitive impact is particularly evident in low‐productivity environments, such as mountain and alpine meadows or nutrient‐poor sites (Hejda 2013). However, large‐statured species like *Arrhenatherum elatius* or ruderal‐adapted species such as *Galium aparine* tend to perform well in *L. polyphyllus* ‐dominated communities (Otte and Maul 2005; Wissman et al. 2015). This may be attributed to the clumped spatial structure of *L. polyphyllus* , which leaves gaps between clusters that other species can exploit (Hejda 2013; Czarniecka‐Wiera et al. 2019). As its cover increases, *L. polyphyllus* has been shown to reduce functional divergence and increase vegetation homogeneity (Hansen et al. 2021). Moreover, its presence alters the community‐weighted means of key functional traits, such as canopy height and LDMC, indicating a shift toward more competitive species (Hansen et al. 2021). Being a legume, *L. polyphyllus* may also enhance soil nitrogen availability (Holdaway and Sparrow 2006), potentially influencing cooccurring species through positive plant–soil feedbacks (Buerdsell et al. 2021). While the broad impacts of *L. polyphyllus* on plant community structure and function are well documented, its specific effects on the functional traits of individual native species remain poorly understood—particularly in light of the contrasting influences of likely beneficial plant–soil interactions and negative, asymmetric competition for light.

In the presented study, we focus on the phenotypic changes of native plants in response to the presence of the invasive *Lupinus polyphyllus* in a community. This study is intended as a mechanistic, species‐level case study focusing on a selected focal species and, therefore, aims to provide insight into underlying processes rather than broad generalizations. Our main aim is to (1) assess the impact of *L. polyphyllus* on the functional traits of native plant species, (2) evaluate whether the presence of *L. polyphyllus* causes a change in the life strategy of native plants, and (3) determine if habitat factors modify these changes. Considering that grasses and herbs differ in terms of anatomical, physiological and ecological characteristics that determine their growth strategy, resource acquisition and stress tolerance (Siebenkäs et al. 2015), we hypothesized that the magnitude of native species' response to the presence of *L. polyphyllus* may vary depending on the species group (grasses vs. herbs). Since the competition for light and nutrients is size‐asymmetric (Weiner 1990; Rajaniemi 2003), it has been observed that in grasslands, the plasticity in response to varying light and nutrients may differ between tall‐ and small‐stature species (Siebenkäs et al. 2015). Therefore, we also tested the effect of plant height on the magnitude of their responses to the presence of *L. polyphyllus* .

## 2 Methods

### 2.1 Study Sites

**FIGURE 1:**
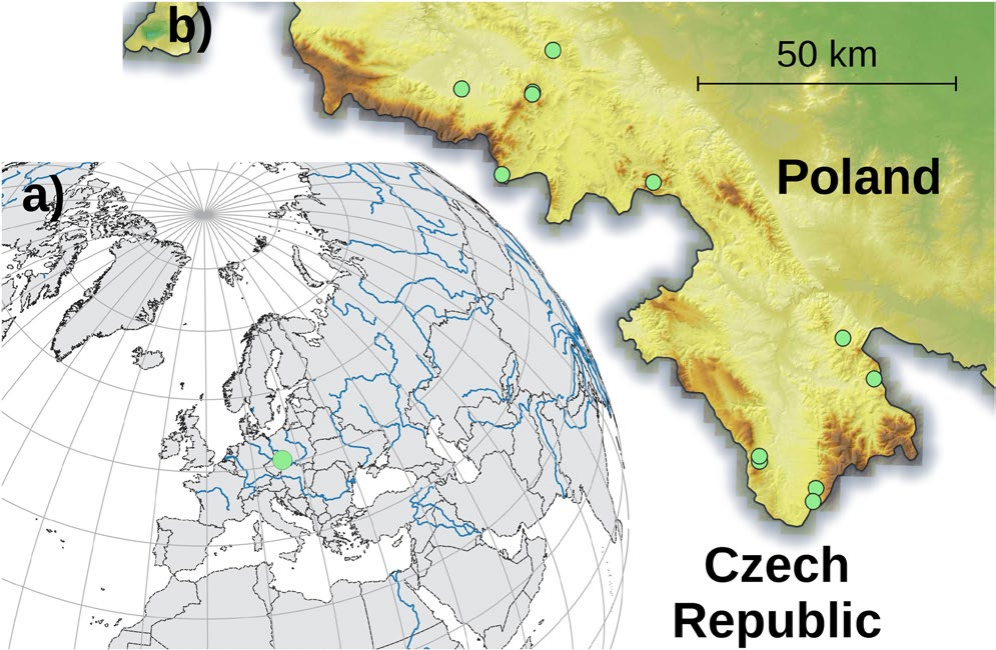
Location of the study area shown against national boundaries (panel a, green dot), and the distribution of study sites over land relief within Poland's borders (panel b, green dots).

The studies were conducted in 2024 in extensively managed, seminatural grasslands located in the Sudetes Mountains and their foreland in SW Poland (Figure 1). The altitude of the study area ranges from 491 to 762 m a.s.l., with a mean annual temperature of 5°C–8°C and annual precipitation between 490 and 650 mm, depending on elevation (Woś 1999). The predominant soil types are acidic brown soils and poorly developed rocky soils, both of which are relatively low in nutrients (Drozd 2015; Table S2 in Data S2 provides detailed data). The seminatural grasslands are classified under the alliances *Arrhenatherion* and *Polygono‐Trisetion* (Kącki et al. 2013).

### 2.2 Trait Data Collection

Initial information on the distribution of *Lupinus polyphyllus* in grasslands was obtained from the Polish Vegetation Database (Kącki and Śliwiński 2012). Based on this data, 12 sites were selected (Figure 1), each with a cover of *L. polyphyllus* exceeding 50%. At each site, two paired plots were established: one dominated by *L. polyphyllus* with Lupinus cover ≥ 50% and a second without *L. polyphyllus* . The distances between plots in each pair ranged from 7 to 30 m (average 18 m), and the stands had to represent the same vegetation type (for details, see Table S2 in Data S2). Each plot was square‐shaped with an area of 10 m^2^ (3.33 × 3.33 m).

In both plots, we collected phytosociological and species trait data, as well as soil samples from the upper soil layer (at a depth of 10 cm). The aboveground biomass was assessed by cutting the plants at a height of 5 cm above ground level in plots sized 1 m^2^, placed in the center of the 10 m^2^ plots. The cover of *L. polyphyllus* in invaded plots ranged from 50% to 98% (with an average of 60%).

In the field, species trait data were collected for eight target species—four grasses ( *Dactylis glomerata* , *Arrhenatherum elatius, Agrostis capillaris, Phleum pratense*) and four herbaceous species (*Hypericum maculatum, Vicia sepium, Veronica chamaedrys, Galium mollugo*). These selected perennial species are characteristic of grassland communities within the *Molinio‐Arrhenatheretea* class (Kącki et al. 2013; Matuszkiewicz 2001) and were among the most frequent in the studied vegetation. For each species, we measured the height of the stem (excluding inflorescences) of five individuals, as some had already lost their flowers. Individuals were randomly selected within the study plot (10 m^2^). From each of the five individuals, we randomly selected five young to medium‐aged, fully expanded leaves. Leaves from each plant were then separated for the calculation of LA and SLA (calculated as leaf area/leaf dry weight). Projected leaf area was determined using a flatbed scanner (WinDIAS) and image analysis software (Delta‐T SCAN; Delta‐T Devices, Cambridge, UK). All leaves were air‐dried and weighed to calculate LDMC. Data collection followed the methodology established in the LEDA database (Kleyer et al. 2008).

### 2.3 Environmental Data

In the soil samples, we evaluated the content of total nitrogen (N), total carbon (C), available phosphorus (P_2_O_5_), available potassium (K_2_O), magnesium (Mg), pH, and granulometry. The soil pH was measured potentiometrically in 1 M KCl. Soil concentrations of plant available potassium (K) and phosphorus (P) were determined using the Egner–Riehm method (Korzeniowska and Stanisławska‐Glubiak 2024). The concentrations of plant‐available magnesium (Mg) were determined using the Schachtschabel method (Schachtschabel 1954). The content of nitrogen and carbon in soil was measured using a dry combustion method. The results of the granulometry analysis were classified according to PTG (2008) classification: coarse fraction (particle diameter above 2 mm), sand (2–0.05 mm), silt (0.05–0.002 mm), and clay (< 0.002 mm in diameter). Based on coordinates of plots measured in the field using a GNSS receiver and digital elevation model with resolution 5 m (DEM), we calculated the altitude (alt), topographic wetness index (TWI), and diurnal anisotropic heating (DAH) (Hengl and Reuter 2008) for each plot. The values of measured and calculated variables are shown in Table S2 in Data S2. To ensure that soil parameters and topographic factors do not differ substantially between invaded and control plots pairs and likely do not correlate with potential shifts in functional traits of native plants' traits, we compared the pairs using GLMM and multivariate PCA analysis (details in Tables S1, S2; Figure S2 in Data S2 and PCA analysis for comparison of soil parameters and topographic factors between control and invaded plots).

### 2.4 Statistical Analysis

Due to the specific data distributions, we decided to employ semi‐parametric and nonparametric tests. All statistical analyses were conducted in the R environment (R Core Team 2023). The effects of the *Lupinus polyphyllus* invasion on plants' functional traits (height, LA, SLA, LDMC) for individual species and functional types (i.e., herbs and grasses) were analyzed using generalized linear mixed models (GLMM) fitted with a template model builder (glmmTMB) in the “glmmTMB” package. Two random effects were included in the model: spatial structure (plots nested within sites) and species identity. The random‐effects structure was specified as (1 | site/plot) + (1 | species). The distribution family was selected based on data type and model diagnostics. The significance of the interactions and main effects was assessed using Wald test statistics. The model's diagnostic was performed using the “DHARMa” package. To define the effect size we calculated the change in the mean value of the dependent variable response, resulting from the change of the predictor value (beta coefficient) multiplied by 100. When log link was applied we used exponence of the change (Brooks et al. 2017). Similarly, generalized linear mixed models (GLMMs) were applied to evaluate the effect of the interaction between *Lupinus polyphyllus* invasion and environmental variables on plant functional traits across species types (i.e., grasses and herbs).

Investment in particular life strategies according to the CSR framework was quantified using the StrateFy tool (Pierce et al. 2017), which determines ecological strategies for target species based on LA, SLA, and LDMC. This yielded CSR ternary coordinates, representing the relative proportional contributions (%) to each life strategy. Differences in these coordinates between invaded and control plots for functional types (i.e., herbs and grasses) and individual species were also evaluated using GLMM models. Overall changes in CSR strategy investment were assessed using the PERMANOVA test, as implemented by the “adonis2” function from the “vegan” package in R. The paired structure of plots was implemented by using the plot's identity with strata(idi). Statistical significance was determined based on 999 permutations.

To test the hypothesis of a correlation between plant height and the magnitude of their response to *L. polyphyllus* presence, Spearman's rank correlation coefficient was calculated between plant height and the effect size for height, leaf area, SLA, and LDMC.

## 3 Results

The control plots hosted a similar number of species as the invaded ones, but the total amount of biomass was higher in the invaded plots (Table S2 in Data S2). The biomass of native species on invaded plots was higher compared to the control if the aboveground productivity per square meter was adjusted by the cover of natives (Figure S2 in Data S2). We did not find significant differences between the invaded and control plots regarding environmental variables (for details, Table S2 in Data S2).

### 3.1 Effect of *L. polyphyllus* on Plant Traits

**FIGURE 2:**
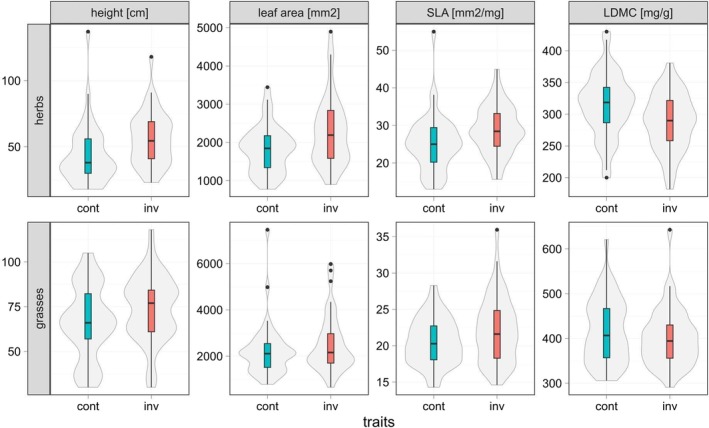
Values of height, leaf area, specific leaf area (SLA), and leaf dry matter content (LDMC) for plots dominated by *Lupinus polyphyllus* (inv, red) and those without invasive species (cont, blue) for herbs (upper panel) and grasses (lower panel). The line represents the median, the box the interquartile range, the whiskers the range of non‐outlying values, and small dots indicate outliers.

We found significant differences in almost all analyzed traits between invaded and control plots for both plant functional types—grasses and herbs (Table 1, Figure 2)—as well as for most individual species (Table S3 in Data S2). Generally, plant height, LA, and SLA values were significantly higher in plots dominated by the invader, while LDMC values were lower compared to control plots (Figure 2). This pattern was observed for most species, particularly *Hypericum maculatum* and *Vicia sepium*, which exhibited the strongest effect size (Table S3; Figure S4 in Data S2). An exception was noted for *Dactylis glomerata*, which did not differ significantly between invaded and control plots (Table S3; Figure S3 in Data S2). Additionally, *Phleum pratense* showed significantly lower LDMC values in invaded plots (Table S3, Figure S3 in Data S2). Higher effect size values for herbs compared to grasses suggest a stronger response of herbs to the presence of *L. polyphyllus* (Table 1 and Table S3 in Data S2, for individual species).

**TABLE 1:** Results of statistical tests (*Z*, *p*) for functional traits (height, leaf area, SLA—specific leaf area and LDMC—leaf dry matter content) between plots invaded by *Lupinus polyphyllus* and control plots for pooled herb and grass species (plant type). The significant differences are highlighted in bold. Additionally shown is the number of observations (N) as well as the effect size.

Plant type	Trait	*N*	*Z*	*p*	Effect size^a^
Herbs	Height	84	4.62	**< 0.001**	33.5
Herbs	Leaf area	84	3.43	**< 0.001**	24.2
Herbs	SLA	84	2.44	**0.01**	14.9
Herbs	LDMC	84	−2.84	**0.004**	−8.3
Grasses	Height	88	2.35	**0.02**	13.4
Grasses	Leaf area	88	2.39	**0.02**	15
Grasses	SLA	88	2.14	**0.03**	7.1
Grasses	LDMC	88	−1.32	0.18	−3.6

^a^ The relative change in % of the mean response.

### 3.2 Importance of Environmental Factors on the Functional Traits of Native Plants

The GLMM models with included interactions between the invader presence and environmental variables revealed that generally, in plots invaded by *Lupinus polyphyllus* , native species responses to environmental factors are weaker than in control plots (Table 2). For herbs, the SLA of native plants in invaded plots increases more slowly with pH and decreases more slowly with clay content than in control plots. Similarly, LDMC of native plants in invaded plots increases more slowly with clay content, while its relationship with pH varies between plot types. The TWI index was positively associated with plant height, with a stronger effect observed in plots without the invasive species. Plant height was also positively related to K_2_O but only in control plots, while the response in invaded plots was minimal (Table 2, Figure S5 in Data S2). For grasses, SLA decreased more slowly with increasing C, N, and C/N content in control plots, whereas LDMC increased more rapidly with higher clay content and DAH index in these plots. In contrast, plant height responded differently to pH depending on the plot type (Table 2, Figure S6 in Data S2).

**TABLE 2:** Results of generalized linear mixed models (GLMM) for the influence of *L. polyphyllos* (type), environmental variables (envir) and interaction between them (interact) on native plants' functional traits (height, leaf area, SLA—specific leaf area and LDMC—leaf dry matter content) calculated for grasses and herbs (plant type), with corresponding statistical test results (*Z*, *p*‐value).

Plant type	Trait	Factor	Type		Envir		Interact		Distribution family
			*z*	*p*	*z*	*p*	*z*	*p*	
Herbs	SLA	pH	2.200	0.03	3.073	0.002	−1.972	0.05	Gamma(log)
	SLA	Clay			−3.353	< 0.001	2.755	0.006	Gamma(log)
	LDMC	pH	−3.569	< 0.001	−2.673	0.007	3.239	0.001	Gamma(log)
	LDMC	Clay	2.423	0.01	3.238	0.001	−3.637	< 0.001	Gamma(log)
	Height	TWI	2.530	0.01	2.884	0.004	−2097	0.03	Gamma(log)
	Height	K2O	4.916	< 0.001	3.249	0.001	−2.607	0.009	Gamma(log)
Grass	SLA	C	2.735	0.006			−2.041	0.04	Gamma(log)
	SLA	N	2.648	0.008			−2.040	0.04	Gamma(log)
	SLA	C/N	2.369	0.02			−2.225	0.03	Gamma(log)
	LDMC	DAH			3.008	0.003	−1.979	0.05	Gamma(log)
	LDMC	Clay			3.095	0.002	−2.492	0.01	Gamma(log)
	Height	pH	2.695	0.007	2.815	0.005	−2.448	0.01	Gamma(log)

### 3.3 Effect of *L. polyphyllus* on the CSR Strategy of Native Plants

Invasion by *L. polyphyllus* resulted in changes in the median CSR strategies of native plants. In control plots, both herbs and grasses predominantly exhibited an S‐selected strategy (Table 3), whereas plants growing in the presence of *L. polyphyllus* showed significantly reduced investment in the S‐strategy, shifting toward greater allocation to the C‐ and R‐strategies (Table 3). Similar patterns were observed for most individual species, except for *Dactylis glomerata* (Table S4 in Data S2).

**TABLE 3:** Median values and changes (delta) in coordinates along the CSR triangle axes (strategy) for grasses and herbs (plant type) in invaded and control plots, with corresponding statistical test results (*Z*, *p*). Bold values represent statistically significant effects (p < 0.05).

Plant type	Strategy	N	*Z*	*p*	Invaded	Control	Delta^a^	Effect size^a^
Herbs	C	84	**4.06**	**< 0.001**	29.881	26.752	3.129	10.4
Herbs	S	84	**−3.61**	**< 0.001**	36.54	43.401	−6.861	−17
Herbs	R	84	**2.42**	**0.01**	32.999	29.464	3.535	15.9
Grasses	C	88	1.79	0.07	26.458	26.399	0.059	5.8
Grasses	S	88	**−2.63**	**0.008**	50.433	53.266	−2.832	−5.3
Grasses	R	88	1.32	0.187	21.495	20.973	0.522	6.6

^a^ Delta = median for invaded plots‐median for control plots.

**FIGURE 3:**
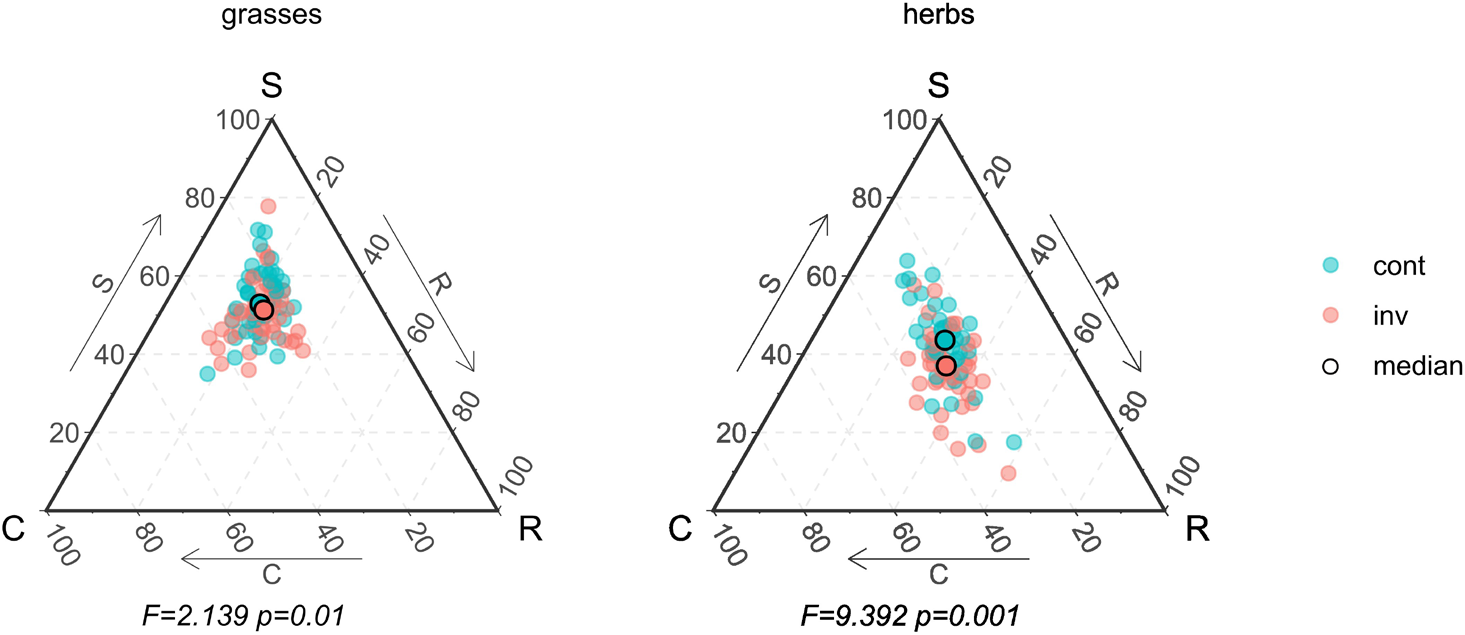
Location of average values for species and plots within the CRS strategy triangle for grasses (left) and herbs (right), along with PERMANOVA results (F and p) comparing CSR strategy differences between native plants in invaded (inv—red points) and control (cont—blue points) plots.

The PERMANOVA results indicate that the presence of invasive *L. polyphyllus* caused a directional shift in the overall percentage investment in strategy types for both grasses and herbs (Figure 3), as well as for individual species (Figures S7 and S8 in Data S2). In invaded plots, herbs occupied the central region of the CSR triangle and exhibited a more generalist strategy, whereas herbs in control plots were clustered near the S‐strategy corner (Figure 3). Similarly, grasses in control plots were concentrated around the S‐strategy corner, but in the presence of *L. polyphyllus* , their distribution shifted slightly toward the C‐ and R‐strategy corners (Figure 3). PERMANOVA tests for individual species revealed comparable patterns, with *Heracleum maculatum* and *Vicia sepium* showing the strongest response, while *Dactylis glomerata* and *Agrostis capillaris* exhibited no significant response to the invader (Figures S7 and S8 in Data S2). The magnitude of change was greater in herbs than in grasses (Table 3, Figure 3).

### 3.4 Correlation Between Plants' Height and Their Reaction to *L. polyphyllus* Invasion

**FIGURE 4:**
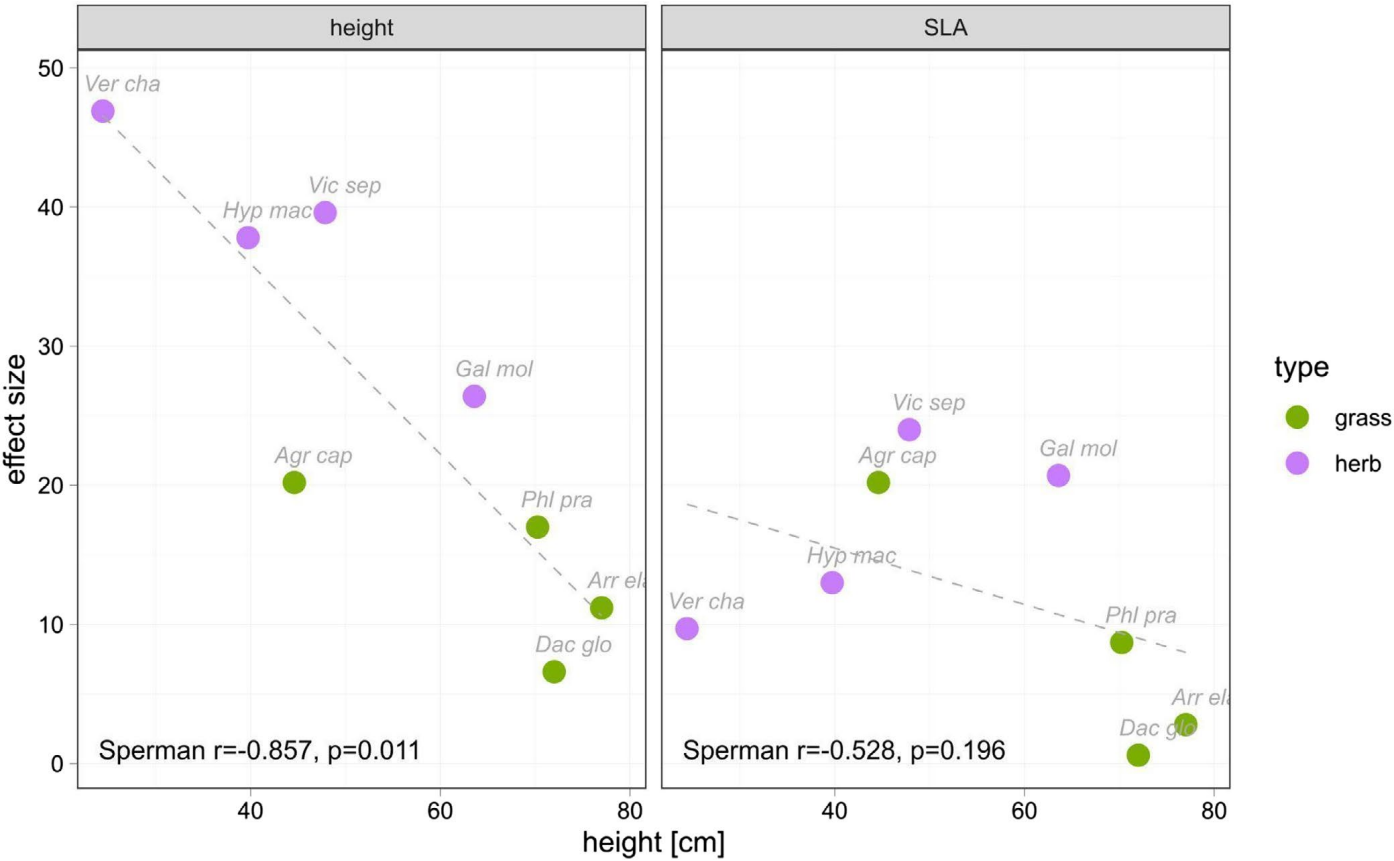
Plant height plotted against effect size for height (left panel) and SLA (specific leaf area—right panel), with fitted linear trend line. Different colors indicate functional types (grasses—green and herbs—violet).

We observed consistent trends in the relationship between plant height and the magnitude of response to the presence of *L. polyphyllus* (Figure 4). However, these results were not statistically significant (Table S5 in Data S2). Shorter‐stature plants tended to exhibit a stronger response compared to taller species (height: *r* = −0.542, *p* = 0.165; SLA: *r* = −0.459, *p* = 0.253).

## 4 Discussion

The directional shift in species traits observed in the present study aligns with the pattern reported by Hansen et al. (2021), who documented community‐level changes during *Lupinus polyphyllus* invasion expressed through community‐weighted means of height, SLA, and LDMC. However, while Hansen et al. (2021) inferred these shifts using trait data from a global database, indicating changes in species composition, our findings demonstrate direct responses of particular species.

### 4.1 The Effect of *L. polyphyllus* and Environmental Gradients on the Species Traits of Native Plants

The observed directional changes in SLA, LDMC, and height related to *L. polyphyllus* presence are symptomatic of decreased light conditions and/or higher soil nutrient availability, relating to increased aboveground productivity. The SLA reflects light acquisition strategies; a greater leaf area per unit mass, achieved through the development of thinner leaves, enhances light capture and is commonly associated with shaded environments (Weiher et al. 2009; Hodgson et al. 2011). Similarly, LDMC tends to increase under higher irradiance and decrease under low light (Hodgson et al. 2011; Bachmann et al. 2018). Consequently, plants growing beneath the canopy of taller neighbors may adjust their morphological traits to cope with reduced light by elongating stems and producing longer, thinner leaves (Valladares and Niinemets 2008; Roscher et al. 2011). Since the *L. polyphyllus* typically reaches about 1.2 m in height and grows in dense clumps, it casts shade on surrounding vegetation (Eckstein et al. 2023). Similar effects of invaders were also observed for other species (Bennett and Klironomos 2019). For example, Dostál et al. (2012) documented enhanced growth of native *Impatiens noli‐tangere* near invasive *Impatiens parviflora* , while Bergum et al. (2012) reported greater vegetative growth of *Sporobolus airoides* in sites invaded by *Acroptilon repens* . Besides the light, the higher soil nutrient availability, and aboveground productivity, increased SLA and decreased LDMC (Hodgson et al. 2011; Smart et al. 2017), often indicating elevated leaf nitrogen content (Hansen et al. 2021). Legumes, such as *L. polyphyllus* , form symbiotic associations with rhizobia bacteria that fix atmospheric nitrogen into forms accessible to plants. This process enriches the soil with nitrogen, thereby enhancing the growth of surrounding vegetation (Barneze et al. 2020); however, we did not record any significant differences in soil properties. A similar lack of differences in nitrogen content between invaded and control plots was found by Meier et al. (2013). It was concluded that *L. polyphyllus* may potentially fertilize soils with a pulse of nitrogen; thus, N fertilization might be observed through higher frequency sampling, along a timeseries, or with isotopic tracers (Meier et al. 2013). In our study, *L. polyphyllus* plots clearly produced higher biomass compared to the controls, considering also native species biomass. It can be explained by the fact that higher SLA and lower LDMC indicate less dense tissues and a shift toward resource‐acquisitive strategies, which may allow native species to maintain comparable biomass despite occupying a smaller area (Wright et al. 2004).

In our study, we also observed differences in reactions on environmental gradients between native plants growing in control plots and those in invaded plots, indicated for species groups (interactions in Table 3 and Figures S5 and S6). It suggests that the *L. polyphyllus* presence can modify the effect of pH, clay content, TWI, and K2O on herbs traits, and likely C, N, C/N, pH, clay content, and DAH on grasses traits. The more gradual increase in SLA and the decrease in LDMC observed in the presence of *L. polyphyllus* along a gradient of rising soil pH can be attributed to *Lupinus polyphyllus* ability to locally reduce soil pH. This buffering effect is likely driven by nitrogen fixation and subsequent nitrification processes (oxidation of NH_4_^+^ to NO_3_^−^), which release hydrogen ions (H^+^) into the soil (Bolan et al. 1991; Andrews and Andrews 2017). Through rhizosphere acidification and the exudation of organic acids *L. polyphyllus* can increase the pool of plant‐available macro‐ and microelements (i.e., K, P, Ca, Mg) and facilitate the growth of neighboring species, particularly in nutrient‐poor or acidic soils (Marschner et al. 1986; Lambers et al. 2013). In our study, we also observed a more gradual decrease in SLA and an increase in LDMC in the presence of *L. polyphyllus* along with increasing soil clay content. This pattern may be related to the species' well‐developed root system, consisting of a taproot and numerous lateral roots, which can influence soil structure. Such root systems may promote the formation of biopores, enhance soil aeration, and locally reduce soil compaction, potentially improving resource availability for plants in highly compacted soils (Eckstein et al. 2023). The presence of *L. polyphyllus* was also associated with a weaker response of SLA to increasing TWI and of LDMC to increasing DAH in native species. Both indices are related to habitat conditions and reflect key habitat gradients: higher TWI values indicate greater soil moisture availability, while higher DAH values correspond to greater solar radiation exposure and diurnal heating, leading to more xerothermic conditions (Kopecký et al. 2021). The presence of a tall, dense canopy formed by *L. polyphyllus* may buffer environmental variability by reducing microclimatic stress, such as fluctuations in moisture and temperature, thereby decreasing habitat xeromorphism (Eckstein et al. 2023). Across C, N, and C/N gradients, SLA responses differed depending on the presence of *L. polyphyllus*. In its absence, SLA increased with increasing nitrogen and carbon and decreased with increasing C/N, reflecting shifts in resource availability consistent with the leaf economics spectrum (Wright et al. 2004). In contrast, in the presence of *L. polyphyllus* , SLA declined across all gradients, suggesting that increasing biomass and canopy development led to stronger light limitation. Under such conditions, competition for light may override the effects of soil resources (Poorter et al. 2009).

### 4.2 The Effect of *Lupinus polyphyllus* on the CSR Strategy of Native Plants

Our results suggest that native plants are capable of modifying their life strategies in response to invasion by alien species. According to Grime (1988), general plant strategies fall into three categories: C (competitors), S (stress‐tolerators), and R (ruderals). However, individual species typically do not conform strictly to a single strategy; instead, they allocate resources among these strategies in varying proportions depending on environmental conditions (Pierce et al. 2017; Hansen et al. 2021). In our study, plants in noninvaded grasslands primarily invested in the S‐strategy, characterized by slow growth and efficient nutrient retention, which enables survival under high‐stress conditions (Grime 1988; Grime and Pierce 2012). Coexistence with invasive legumes appears to be associated with altered responses to certain stressors—most likely some factors limiting biomass productivity—allowing a shift toward greater investment in competitive (C) and ruderal (R) strategies. This suggests that, at the studied sites, the shading stress from *L. polyphyllus* is less limiting than the nutrient stress.

### 4.3 The Reaction of Herbs vs. Grasses on the *L. polyphyllus* Invasion

It has been observed that taller species tend to benefit, while low‐stature plants are disadvantaged in the presence of *Lupinus polyphyllus* due to asymmetric light competition (DeMalach et al. 2017; Eckstein et al. 2023). Nonetheless, in our study, small herbs such as *Galium mollugo, Veronica chamaedrys*, and *Vicia sepium* appeared to be less affected by this stress. Generally, grass species exhibit trait combinations indicative of more conservative resource use, while herbs are characterized by traits associated with a more exploitative strategy (Siebenkäs et al. 2015). It has been hypothesized that exploitative species display greater trait plasticity than conservative ones in response to increased nutrient availability (Crick and Grime 1987). Additionally, short‐stature species tend to exhibit greater trait variation in response to changes in light availability compared to taller species (Siebenkäs et al. 2015). In our dataset, herb species were generally shorter than grasses. Therefore, the stronger response observed in herbs likely results from both inherent differences between plant functional types and stature‐related variation. Overall, the trends observed in our study indicate a stronger response in short‐stature species. However, due to sample size limitations, this correlation was not statistically significant, as the analysis included a limited number of species representing different functional types.

### 4.4 Limitations of the Study

The results presented were derived from 8 species and 12 study sites. The limitations result from time‐consuming trait measurements of individuals. Moreover, the measurements must be conducted within a reasonably short time period to minimize the effect of differences in phenological development among the studied plants between the analyzed sites. Additionally, we aim to find the same species at all sampled sites and plots, which also restricts the number of sites. Therefore, the observed effect must be relatively strong to be statistically significant. Particularly observed here, the effect of soil properties was significant only for species types (herbs and grasses), but not for particular species, likely due to the limited number of observations. Also, the tendency of short‐stature species to have a stronger reaction to invasive *Lupinus polyphyllus* (Figure 4) is not statistically significant, perhaps due to the limited number of species examined, which included a mix of different functional types (i.e., grasses and herbs). It is also suggested that the pulse of nitrogen soil fertilization by *Lupinus polyphyllus* is short‐lived; thus, a more intensive field sampling frequency is required to capture it. In our case, the soils were sampled once per plot in mid‐vegetation season; thus, it is likely that we missed the pulse, resulting in a lack of soil properties between invaded and control plots, in addition to the observed effects on plants. The other limitation of this study is that light availability was not directly measured at ground level. Such measurements could have provided more detailed insight into how *L. polyphyllus* influences resource availability, particularly through shading effects, and helped to better disentangle the relative importance of light versus other environmental factors. Finally, the studied grassland has developed on an acidic and strongly acidic soil substrate (pH range: 4.0–5.2, average 4.3; for details, see Table S2); it is unclear how the *L. polyphyllus* effect will be on less acidic soils.

### 4.5 Conclusions

Our study shows that native plants growing in grasslands invaded by *Lupinus polyphyllus* are generally taller, with larger leaf areas, higher SLA, and lower LDMC than those in noninvaded grasslands (Figures S9 and S10 in Data S2). The observed modifications in leaf traits among plants interacting with the invasive species suggest a shift away from stress‐tolerant strategies, with a corresponding increase in investment in competitive and ruderal strategies. This shift may result from plant—soil interactions that modify the effects of low nutrient availability, and the reduced light availability, prompting native species to adjust their morphology to cope with low‐light conditions. The impact of *L. polyphyllus* invasion on functional traits was more pronounced in herbs than in grasses. This likely reflects inherent differences between the groups: grasses tend to follow a conservative resource‐use strategy, whereas herbs exhibit traits associated with a more exploitative strategy. As a result, herbs may be better able to adapt and coexist with the invasive species. From a practical perspective, our findings suggest that incorporating herbs (e.g., in seed mixtures) may be more effective than using grasses alone when restoring grasslands invaded by *Lupinus polyphyllus* . These findings suggest that trait‐based approaches may serve as an early‐warning indicator of shifts in plant community functioning and could support management strategies aimed at mitigating the impacts of *L. polyphyllus* invasion.

## Author Contributions

**Marta Czarniecka‐Wiera:** conceptualization (equal), data curation (equal), formal analysis (equal), funding acquisition (lead), investigation (equal), methodology (equal), project administration (lead), software (equal), visualization (supporting), writing – original draft (equal), writing – review and editing (equal). **Boglárka Berki:** conceptualization (supporting), data curation (equal), investigation (equal), methodology (equal), resources (equal), software (equal), writing – original draft (supporting), writing – review and editing (supporting). **Tomasz H. Szymura:** conceptualization (equal), formal analysis (equal), methodology (equal), software (equal), visualization (lead), writing – original draft (equal), writing – review and editing (equal). **Magdalena Szymura:** conceptualization (equal), methodology (equal), resources (equal), supervision (lead), validation (lead), writing – original draft (equal), writing – review and editing (equal).

## Funding

This work was supported by the Wrocław University of Environmental and Life Sciences (Poland) as part of the research project no N060/0007/24.

## Conflicts of Interest

The authors declare no conflicts of interest.

## Supporting information


**Data S1:** ece373911‐sup‐0001‐DataS1.xlsx.

**Data S2:** ece373911‐sup‐0002‐Supinfo.zip.
**Table S1:** Geographical coordinates, spatial distance between paired plots at a site, species richness, aboveground biomass, and values of environmental variables for paired plots with results of statistical tests of differences between invaded and control plots (Chi^2^, p and distribution family). The abbreviations of variable names: N species—species richness, biomass—aboveground biomass, alt—altitude, TWI—topographic wetness index, DAH—diurnal anisotropic heating, N‐total nitrogen, C—total carbon, P—available phosphorus (P2O5), K—available potassium (K2O), Mg—available magnesium, pH—soil pH measured in a 1 M KCl solution, coarse—coarse soil fraction (particle diameter above 2 mm), sand—sand fraction (2–0.05 mm), silt—silt fraction (0.05–0.002 mm), and clay—clay fraction (< 0.002 mm in diameter).
Multivariate PCA analysis for comparison of soil parameters and topographic factors between control and invaded plots.
**Table S2:** Values of loadings and explained variance in PCA analysis of environmental variables. The loadings with highest value in particular PCA axis are bolded. Variable names abbreviation the same as in Table S2.
**Table S3:** Results of statistical tests (Z, p, effect size) for functional traits between plots invaded by *Lupinus polyphyllus* and control plots for particular species (species). The significant differences are highlighted in bold. Additionally shown is the affinity of a species to plant functional types (plant type), number of observed pairs (N), as well as effect size.
**Table S4:** Median values and changes (delta) in coordinates along the CSR triangle axes (strategy) for target species (species) in invaded and control plots with corresponding statistical test results (Z, p). Bolding letters indicate significant differences.
**Table S5:** Spearman rank correlation matrix (r—upper triangle, p—lower triangle) among median of species height and effect size (ef) for height (ef‐height), leaf dry matter content (ef_LDMC), leaf area (ef_LA) and specific leaf area (ef_SLA).
**Figure S1:** Results of PCA analysis (main panel) and summary of distances in multivariate PCA space for paired plots and sites (small, upper panel).
**Figure S2:** Comparison of the amount of biomass in the control (cont.) and invaded (inv.) plots: (a) biomass content in 1m^2^, (b) biomass content considering the coverage of native species. Note the different y‐axis scales.
**Figure S3:** Values of height, leaf area, specific leaf area (SLA), and leaf dry matter content (LDMC) for plots dominated by *Lupinus polyphyllus* (inv. red) and those without invasive species (cont. blue) for grass species. The line represents the median, the box the interquartile range, the whiskers the range of non‐outlying values, and small dots indicate outliers.
**Figure S4:** Values of height, leaf area, specific leaf area (SLA), and leaf dry matter content (LDMC) for plots dominated by *Lupinus polyphyllus* (inv. red) and those without invasive species (cont. blue) for herbs. The line represents the median, the box the interquartile range, the whiskers the range of non‐outlying values, and small dots indicate outliers.
**Figure S5:** Values of species traits for herbs versus environmental variables for plots dominated by *Lupinus polyphyllus* (inv. red) and those without invasive species (cont. blue) for herbs.
**Figure S6:** Values of species traits for grasses versus environmental variables for plots dominated by *Lupinus polyphyllus* (inv. red) and those without invasive species (cont. blue) for herbs.
**Figure S7:** Location of average values for species and plots within the CRS strategy triangle for grass species along with PERMANOVA results (F and p) comparing CSR strategy differences between plants in invaded (red points) and control (blue points) plots.
**Figure S8:** Location of average values for species and plots within the CRS strategy triangle for herbs along with PERMANOVA results (F and p) comparing CSR strategy differences between plants in invaded (red points) and control (blue points) plots.
**Figure S9:** Landscape of mountain grasslands invaded by *Lupinus polyphyllus* (upper panel) and adjacent grasslands without invasive species (bottom panel) (photo by Boglárka Berki, 25.06.2024).
**Figure S10:** Comparison of the native plants' size grown in grasslands invaded by *Lupinus polyphyllus* (left plant) and without invasive species (right plant) (photo by Marta Czarniecka‐Wiera, 25.06.2024).

## Data Availability

The data that supports the findings of this study are available in the Supporting Information of this article.
