# Supplementary material for: Invasive Lupinus polyphyllus Alters Functional Traits and Life Strategies of Native Species
Source: Ecol Evol. 2026 Jun 29;16(7):e73911. doi: 10.1002/ece3.73911 (PMC13314384; doi:10.1002/ece3.73911)
Supplement: Supplementary file 2 — Data S2: ece373911‐sup‐0002‐Supinfo.zip. Table S1: Geographical coordinates, spatial distance between paired plots at a site, species richness, aboveground biomass, and values of environmental variables for paired plots with results of statistical tests of differences between invaded and control plots (Chi2, p and distribution family). The abbreviations of variable names: N species—species richness, biomass—aboveground biomass, alt—altitude, TWI—topographic wetness index, DAH—diurnal anisotropic heating, N‐total nitrogen, C—total carbon, P—available phosphorus (P2O5), K—available potassium (K2O), Mg—available magnesium, pH—soil pH measured in a 1 M KCl solution, coarse—coarse soil fraction (particle diameter above 2 mm), sand—sand fraction (2–0.05 mm), silt—silt fraction (0.05–0.002 mm), and clay—clay fraction (< 0.002 mm in diameter). Multivariate PCA analysis for comparison of soil parameters and topographic factors between control and invaded plots. Table S2: Values of loadings and explained variance in PCA analysis of environmental variables. The loadings with highest value in particular PCA axis are bolded. Variable names abbreviation the same as in Table S2. Table S3: Results of statistical tests (Z, p, effect size) for functional traits between plots invaded by Lupinus polyphyllus and control plots for particular species (species). The significant differences are highlighted in bold. Additionally shown is the affinity of a species to plant functional types (plant type), number of observed pairs (N), as well as effect size. Table S4: Median values and changes (delta) in coordinates along the CSR triangle axes (strategy) for target species (species) in invaded and control plots with corresponding statistical test results (Z, p). Bolding letters indicate significant differences. Table S5: Spearman rank correlation matrix (r—upper triangle, p—lower triangle) among median of species height and effect size (ef) for height (ef‐height), leaf dry matter content (ef_LD [file ECE3-16-e73911-s001.zip › Supplementary_material_clean version.docx]

Title: ***Lupinus polyphyllus* alters functional traits and life strategies of native species**

**Supplementary material**

**Appendix 1**

1. **Table S1**. Geographical coordinates, spatial distance between paired plots at a site, species richness, aboveground biomass, and values of environmental variables for paired plots with results of statistical tests of differences between invaded and control plots (Chi^2^, p and distribution family). The abbreviations of variable names: N species - species richness, biomass - aboveground biomass, alt – altitude, TWI - topographic wetness index, DAH - diurnal anisotropic heating, N-total nitrogen, C - total carbon, P - available phosphorus (P2O5), K - available potassium (K2O), Mg - available magnesium, pH - soil pH measured in a 1 M KCl solution, coarse - coarse soil fraction (particle diameter above 2 mm), sand – sand fraction (2-0.05 mm), silt – silt fraction (0.05-0.002 mm), and clay – clay fraction (<0.002 mm in diameter).
2. Multivariate PCA analysis for comparison of soil parameters and topographic factors between control and invaded plots.
3. **Table S2**. Values of loadings and explained variance in PCA analysis of environmental variables. The loadings with highest value in particular PCA axis are bolded. Variable names abbreviation the same as in Table S1.
4. **Fig S1.** Results of PCA analysis (main panel) and summary of distances in multivariate PCA space for paired plots and sites (small, upper panel).
5. **Fig S2.** Comparison of the amount of biomass in the control (cont.) and invaded (inv.) plots: a) biomass content in 1m², b) biomass content considering the coverage of native species. Note the different y-axis scales.
6. **Table S3.** Results of statistical tests (Z, p, effect size) for functional traits between plots invaded by *Lupinus polyphyllus* and control plots for particular species (species). The significant differences are highlighted in bold. Additionally shown is the affinity of a species to plant functional types (plant type), number of observed pairs (N), as well as effect size.
7. **Figure S3.** Values of height, leaf area, specific leaf area (SLA), and leaf dry matter content (LDMC) for plots dominated by *Lupinus polyphyllus* (inv. red) and those without invasive species (cont. blue) for grass species. The line represents the median, the box the interquartile range, the whiskers the range of non-outlying values, and small dots indicate outliers.
8. **Figure S4** Values of height, leaf area, specific leaf area (SLA), and leaf dry matter content (LDMC) for plots dominated by *Lupinus polyphyllus* (inv. red) and those without invasive species (cont. blue) for herbs. The line represents the median, the box the interquartile range, the whiskers the range of non-outlying values, and small dots indicate outliers.
9. **Figure S5.** Values of species traits for herbs versus environmental variables for plots dominated by *Lupinus polyphyllus* (inv. red) and those without invasive species (cont. blue) for herbs.
10. **Figure S6.** Values of species traits for grasses versus environmental variables for plots dominated by *Lupinus polyphyllus* (inv. red) and those without invasive species (cont. blue) for herbs.
11. **Table S4.** Median values and changes (delta) in coordinates along the CSR triangle axes (strategy) for target species (species) in invaded and control plots with corresponding statistical test results (Z, p). Bolding letters indicate significant differences.
12. **Figure S7.** Location of average values for species and plots within the CRS strategy triangle for grass species along with PERMANOVA results (F and p) comparing CSR strategy differences between plants in invaded (red points) and control (blue points) plots.
13. **Figure S8.** Location of average values for species and plots within the CRS strategy triangle for herbs along with PERMANOVA results (F and p) comparing CSR strategy differences between plants in invaded (red points) and control (blue points) plots.
14. **Table S5.** Spearman rank correlation matrix (r - upper triangle, p - lower triangle) among median of species height and effect size (ef) for height (ef-height), leaf dry matter content (ef_LDMC), leaf area (ef_LA) and specific leaf area (ef_SLA).
15. **Figure S9.** Landscape of mountain grasslands invaded by *Lupinus polyphyllus* (upper panel) and adjacent grasslands without invasive species (bottom panel) (photo by Boglárka Berki, 25.06.2024).
16. **Figure S10.** Comparison of the native plants' size grown in grasslands invaded by *Lupinus polyphyllus* (left plant) and without invasive species (right plant) (photo by Marta Czarniecka-Wiera, 25.06.2024).

**Table S1**. Geographical coordinates, spatial distance between paired plots at a site, species richness, aboveground biomass, and values of environmental variables for paired plots with results of statistical tests of differences between invaded and control plots (Chi^2^, p and distribution family). The abbreviations of variable names: N species - species richness, biomass - aboveground biomass, alt – altitude, TWI - topographic wetness index, DAH - diurnal anisotropic heating, N-total nitrogen, C - total carbon, P - available phosphorus (P_2_O_5_), K - available potassium (K_2_O), Mg - available magnesium, pH - soil pH measured in a 1 M KCl solution, coarse - coarse soil fraction (particle diameter above 2 mm), sand – sand fraction (2-0.05 mm), silt – silt fraction (0.05-0.002 mm), and clay – clay fraction (<0.002 mm in diameter).

| site | group | Longitude | Latitude | distance [m] | cover* [%] | | N species | biomass [g/m^2^] | alt [m. a.s.l.] | TWI | DAH | C [%] | N [%] | C/N | P2O5 [mg/100g] | K2O [mg/100g] | Mg [mg/100g] | pH | coarse [%] | sand [%] | silt [%] | clay [%] |
| --- | --- | --- | --- | --- | --- | --- | --- | --- | --- | --- | --- | --- | --- | --- | --- | --- | --- | --- | --- | --- | --- | --- |
|  |  |  |  |  | total | *Lupinus* |  |  |  |  |  |  |  |  |  |  |  |  |  |  |  |  |
| 3 | control | 16.807973 | 50.426031 | 7.0 | 101 | 0 | 21 | 383.7 | 509.9 | 10.85 | 0.08 | 3.77 | 0.34 | 11.00 | 5.1 | 9.4 | 11.5 | 4.2 | 15 | 62 | 34 | 4 |
| 3 | invaded | 16.808000 | 50.426045 |  | 128 | 70 | 25 | 583.0 | 510.2 | 10.85 | 0.08 | 3.03 | 0.28 | 10.72 | 5.4 | 20.1 | 12.2 | 4.0 | 24 | 63 | 32 | 5 |
| 5 | control | 16.895616 | 50.357062 | 22.1 | 118 | 0 | 22 | 480.3 | 514.3 | 10.46 | 0.06 | 2.13 | 0.22 | 9.73 | 1.0 | 8.1 | 41.4 | 5.2 | 18 | 37 | 60 | 3 |
| 5 | invaded | 16.895612 | 50.357261 |  | 121 | 50 | 28 | 716.1 | 519.2 | 10.45 | 0.05 | 3.50 | 0.33 | 10.74 | 1.0 | 5.8 | 26.0 | 5.2 | 26 | 37 | 61 | 2 |
| 10 | control | 16.747344 | 50.164616 | 7.2 | 93 | 0 | 24 | 213.2 | 713.1 | 10.75 | 0.04 | 3.49 | 0.30 | 11.65 | 4.3 | 4.6 | 1.0 | 4.1 | 63 | 52 | 43 | 5 |
| 10 | invaded | 16.747351 | 50.164551 |  | 126 | 85 | 24 | 762.7 | 713.1 | 10.75 | 0.04 | 3.17 | 0.29 | 10.85 | 3.0 | 3.3 | 11.1 | 4.1 | 40 | 57 | 38 | 5 |
| 12 | control | 16.740558 | 50.141405 | 17.9 | 106 | 0 | 20 | 397.7 | 606.9 | 10.44 | 0.12 | 2.55 | 0.25 | 10.21 | 1.0 | 9.5 | 7.3 | 4.5 | 18 | 46 | 50 | 4 |
| 12 | invaded | 16.740666 | 50.141550 |  | 122 | 55 | 26 | 520.4 | 608.9 | 10.00 | 0.17 | 2.56 | 0.25 | 10.45 | 1.9 | 7.3 | 7.3 | 4.4 | 29 | 52 | 44 | 4 |
| 13 | control | 16.592922 | 50.208499 | 16.9 | 108 | 0 | 26 | 305.86 | 739.3 | 9.78 | -0.08 | 2.85 | 0.24 | 11.81 | 1.0 | 5.6 | 5.7 | 4.3 | 42 | 51 | 46 | 3 |
| 13 | invaded | 16.592903 | 50.208348 |  | 120 | 80 | 24 | 631.7 | 740.3 | 9.78 | -0.08 | 3.53 | 0.30 | 11.89 | 1.0 | 4.5 | 6.0 | 4.4 | 45 | 52 | 45 | 3 |
| 14 | control | 16.592196 | 50.217388 | 20.0 | 123 | 0 | 32 | 403 | 719.4 | 12.18 | -0.07 | 3.84 | 0.34 | 11.18 | 1.0 | 6.4 | 2.8 | 4.3 | 59 | 53 | 42 | 5 |
| 14 | invaded | 16.592185 | 50.217208 |  | 137 | 75 | 28 | 581.2 | 720 | 11.19 | -0.11 | 3.46 | 0.30 | 11.67 | 1.6 | 3.2 | 1.5 | 4.0 | 31 | 54 | 42 | 4 |
| 21 | control | 15.991333 | 50.906722 | 21.7 | 96 | 0 | 28 | 135.8 | 491.6 | 9.75 | 0.22 | 4.08 | 0.32 | 12.67 | 3.1 | 10.5 | 7.1 | 4.2 | 12 | 33 | 62 | 5 |
| 21 | invaded | 15.991028 | 50.906750 |  | 101 | 90 | 26 | 337.6 | 491.1 | 9.35 | 0.24 | 3.01 | 0.26 | 11.69 | 3.4 | 13.5 | 6.9 | 4.1 | 6 | 26 | 66 | 8 |
| 24 | control | 15.940333 | 50.833111 | 12.9 | 104 | 0 | 27 | 300.3 | 754.8 | 12.32 | -0.14 | 4.28 | 0.41 | 10.48 | 1.8 | 17.5 | 11.9 | 4.1 | 17 | 45 | 52 | 3 |
| 24 | invaded | 15.940274 | 50.833221 |  | 100 | 90 | 31 | 520.3 | 754.7 | 12.32 | -0.14 | 4.02 | 0.36 | 11.24 | 1.9 | 15.6 | 12.7 | 4.1 | 12 | 48 | 49 | 3 |
| 25 | control | 15.939286 | 50.829385 | 30.8 | 101 | 0 | 19 | 292.0 | 802.7 | 10.85 | -0.16 | 6.22 | 0.52 | 11.96 | 3.8 | 10.1 | 10.1 | 4.0 | 25 | 51 | 46 | 3 |
| 25 | invaded | 15.939442 | 50.829126 |  | 110 | 75 | 19 | 492.1 | 804.3 | 10.84 | -0.12 | 6.54 | 0.55 | 11.84 | 4.0 | 11.1 | 7.2 | 4.0 | 28 | 47 | 48 | 5 |
| 27 | control | 15.865927 | 50.687993 | 12.4 | 110 | 0 | 39 | 117.5 | 792.2 | 9.95 | -0.25 | 4.78 | 0.41 | 11.79 | 1.9 | 12.6 | 9.3 | 4.0 | 33 | 59 | 37 | 4 |
| 27 | invaded | 15.866056 | 50.687917 |  | 102 | 98 | 19 | 654.2 | 792.3 | 10.30 | -0.25 | 6.42 | 0.52 | 12.24 | 3.6 | 10.7 | 7.4 | 4.0 | 38 | 53 | 43 | 4 |
| 32 | control | 15.745731 | 50.833585 | 22.4 | 74 | 0 | 16 | 225.1 | 463.8 | 9.91 | -0.06 | 3.31 | 0.31 | 10.81 | 4.7 | 12.3 | 8.4 | 4.0 | 31 | 61 | 36 | 3 |
| 32 | invaded | 15.745967 | 50.833451 |  | 96 | 80 | 15 | 445.6 | 462.4 | 10.15 | -0.05 | 4.05 | 0.34 | 11.93 | 4.2 | 12.7 | 11.6 | 4.5 | 10 | 55 | 41 | 4 |
| 34 | control | 16.278939 | 50.685108 | 22.2 | 104 | 0 | 16 | 564.6 | 794.2 | 11.00 | -0.04 | 8.07 | 0.62 | 13.00 | 16.4 | 29.1 | 16.0 | 5.1 | 46 | 56 | 42 | 2 |
| 34 | invaded | 16.279250 | 50.685083 |  | 101 | 80 | 16 | 570.9 | 795.2 | 10.52 | -0.06 | 9.81 | 0.76 | 12.89 | 12.7 | 28.6 | 14.9 | 4.7 | 59 | 55 | 42 | 3 |
|  |  |  |  |  |  |  |  |  |  |  |  |  |  |  |  |  |  |  |  |  |  |  |
|  | Chi2 |  |  |  |  | - | 0.148 | 33.540 | 3.255 | 2.007 | 0.630 | 1.464 | 1.112 | 0.759 | 0.099 | 0.032 | 0.164 | 0.416 | 0.451 | 0.237 | 0.900 | 2.056 |
|  | p |  |  |  |  | - | 0.707 | <0.000 | 0.071 | 0.156 | 0.428 | 0.226 | 0.304 | 0.384 | 0.753 | 0.955 | 0.685 | 0.519 | 0.500 | 0.627 | 0.976 | 0.152 |
|  | distribution |  |  |  |  | - | Poisson | Gausiann | Gausiann | Gausiann | Gausiann | Gausiann | Gausiann | Gausiann | Gausiann | Gausiann | Gaussian | Gamma | Gamma | Gaussian | Gamma | Gamma |

* cover [%] on 1m^2^ plots

**2) Multivariate PCA analysis for comparison of soil parameters and topographic factors between control and invaded plots.**

Before performing the PCA we conducted Bartlett’s sphericity test whose results (χ² = 3488, p < 0.001) indicated that the data were suitable for analysis. The data were then scaled and centred and a PCA was performed using bootstrapped PCA with the MultBiplotR package (Vicente-Villardon et al. 2023) in the R environment. The results are presented in **Table S2** and in **Fig. S1** (large panel).

To quantify the magnitude of differences between plots versus variability among sites, we calculated average distances between pairs of plots from the same site in the multivariate PCA space of environmental conditions. Similarly, differentiation among sites, where the site location in the PCA space was calculated as an average value for the two plots (control and invaded) for a particular site. The results show that the distances among sites are approximately six times larger compared to the distances among plots within a site (**Fig. S1**, smaller panel).

To confirm the lack of differences between invaded and control plots at particular sites, the PERMANOVA test was performed with an assumed paired design of plots. In the PERMANOVA, we used ‘manhatan’ distance on scaled and centered environmental data. The results did not reveal significant differences between invaded and uninvaded plots (F = 0.1326. p = 0.572).

**Table S2**. Values of loadings and explained variance in PCA analysis of environmental variables. The loadings with highest value in particular PCA axis are bolded. Variable names abbreviation the same as in Table S1.

| variable | **PCA 1** | **PCA 2** | **PCA 3** |
| --- | --- | --- | --- |
| alt | **0.302** | 0.096 | -0.009 |
| TWI | 0.124 | 0.067 | **-0.212** |
| DAH | **-0.268** | -0.135 | 0.136 |
| C | **0.375** | -0.164 | 0.079 |
| N | **0.374** | -0.157 | 0.046 |
| C/N | **0.275** | -0.087 | 0.249 |
| P2O5 | **0.289** | -0.181 | 0.075 |
| K2O | 0.237 | **-0.242** | -0.009 |
| Mg | -0.046 | **-0.284** | -0.234 |
| pH | -0.026 | **-0.286** | -0.131 |
| coarse | **0.231** | 0.145 | 0.032 |
| sand | 0.227 | **0.286** | -0.107 |
| silt | -0.212 | **-0.307** | 0.074 |
| clay | -0.143 | 0.111 | **0.265** |
|  |  |  |  |
| explained variance [%] | 38.20 | 21.37 | 14.78 |
| cumulative explained variance [%] | 38.20 | 59.57 | 74.35 |

**Fig S1**. Results of PCA analysis (main panel), and summary of distances in multivariate PCA space for paired plots and sites (small, upper panel).

**
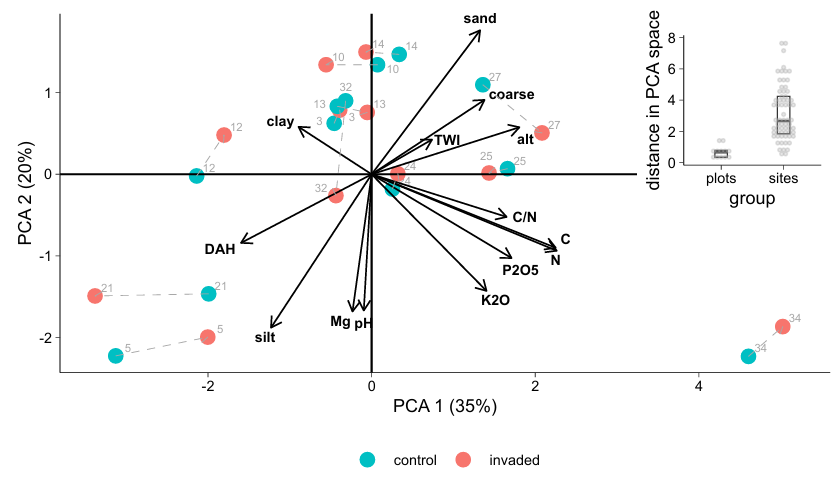
**

**References**

Vicente-Villardon JL, Vicente-Gonzalez L, Frutos-Bernal E (2023) MultBiplotR: Multivariate Analysis Using Biplots in R_. https://doi.org/10.32614/CRAN.package.MultBiplotR>. R package version 23.11.0.

**Figure S2.** Comparison of the amount of biomass in the control (cont) and invaded (inv) plots: a) biomass content in 1m², b) biomass content considering the coverage of native species. Note the different y-axis scales.


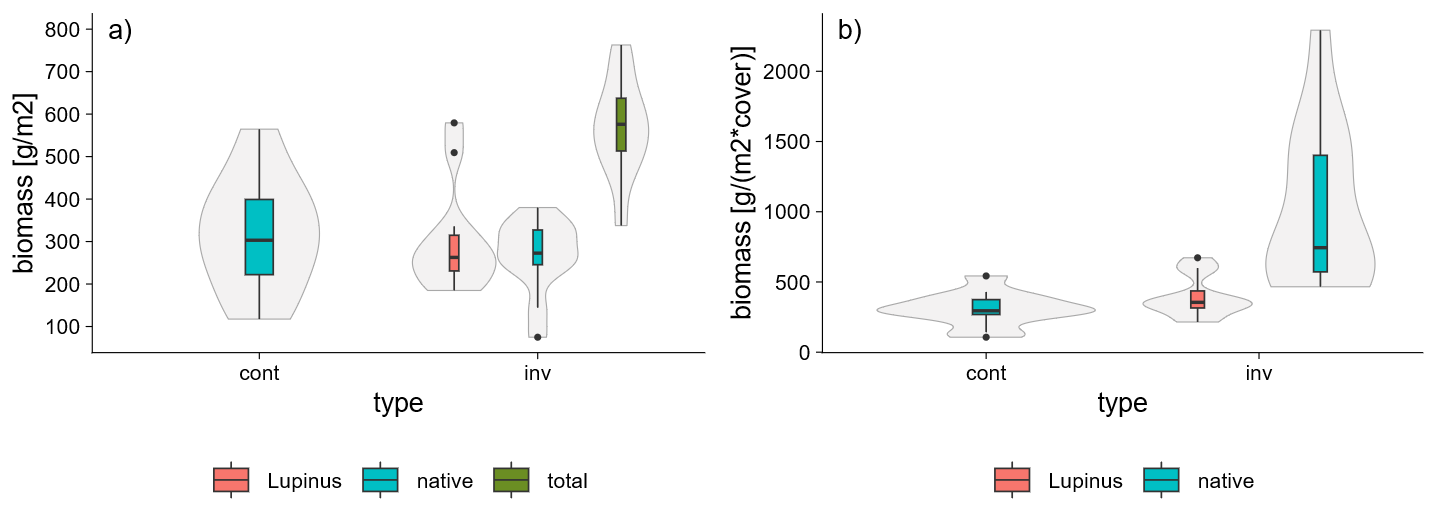


**Table S3.** Results of statistical tests (Z, p, effect size) for functional traits between plots invaded by *Lupinus polyphyllus* and control plots for particular species (species). The significant differences are highlighted in bold. Additionally shown is the affinity of a species to plant functional types (plant type), total number of observations (N), as well as effect size.

| **species** | **plant type** | **trait** | ***N*** | ***Z*** | ***p*** | **effect size*** |
| --- | --- | --- | --- | --- | --- | --- |
| *Agrostis capillaris* | grass | height [cm] | 20 | 1.73 | 0.08 | 22.2 |
| *Agrostis capillaris* | grass | leaf area [mm^2^] | 20 | 1.07 | 0.283 | 13.8 |
| *Agrostis capillaris* | grass | SLA [mm^2^/mg] | 20 | **2.13** | **0.03** | **17.8** |
| *Agrostis capillaris* | grass | LDMC [mg/g] | 20 | 0.22 | 0.829 | 1.3 |
| *Arrhetherum elatius* | grass | height [cm] | 20 | **2.70** | **0.007** | **11.2** |
| *Arrhetherum elatius* | grass | leaf area [mm^2^] | 20 | **2.07** | **0.04** | **17.7** |
| *Arrhetherum elatius* | grass | SLA [mm^2^/mg] | 20 | 0.97 | 0.33 | 2.8 |
| *Arrhetherum elatius* | grass | LDMC [mg/g] | 20 | **-3.44** | **<0.001** | **-9.1** |
| *Dactylis glomerata* | grass | height [cm] | 24 | 0.92 | 0.359 | 6.1 |
| *Dactylis glomerata* | grass | leaf area [mm^2^] | 24 | 0.07 | 0.947 | 0.9 |
| *Dactylis glomerata* | grass | SLA [mm^2^/mg] | 24 | 0.09 | 0.932 | 0.5 |
| *Dactylis glomerata* | grass | LDMC [mg/g] | 24 | 0.25 | 0.799 | 1.3 |
| *Galium mollugo* | herb | height [cm] | 24 | **2.18** | **0.03** | **26.6** |
| *Galium mollugo* | herb | leaf area [mm^2^] | 24 | **2.49** | **0.01** | **29.2** |
| *Galium mollugo* | herb | SLA [mm^2^/mg] | 24 | 1.82 | 0.07 | 19.3 |
| *Galium mollugo* | herb | LDMC [mg/g] | 24 | -1.44 | 0.15 | -6.9 |
| *Hypericum maculatum* | herb | height [cm] | 22 | **2.51** | **0.01** | **26.8** |
| *Hypericum maculatum* | herb | leaf area [mm^2^] | 22 | **2.16** | **0.03** | **18.3** |
| *Hypericum maculatum* | herb | SLA [mm^2^/mg] | 22 | **2.12** | **0.03** | **13.1** |
| *Hypericum maculatum* | herb | LDMC [mg/g] | 22 | **-4.08** | **<0.001** | **-9.7** |
| *Phleum pratense* | grass | height [cm] | 24 | 1.25 | 0.213 | 13.9 |
| *Phleum pratense* | grass | leaf area [mm^2^] | 24 | **2.61** | **0.009** | **25.5** |
| *Phleum pratense* | grass | SLA [mm^2^/mg] | 24 | 1.88 | 0.06 | 8.6 |
| *Phleum pratense* | grass | LDMC [mg/g] | 24 | **-1.87** | 0.06 | **-7.5** |
| *Veronica chamaedrys* | herb | height [cm] | 24 | **5.33** | **<0.001** | **47** |
| *Veronica chamaedrys* | herb | leaf area [mm^2^] | 24 | **4.45** | **<0.001** | **30.5** |
| *Veronica chamaedrys* | herb | SLA [mm^2^/mg] | 24 | 1.31 | 0.19 | 9.7 |
| *Veronica chamaedrys* | herb | LDMC [mg/g] | 24 | -0.63 | 0.527 | -3 |
| *Vicia sepium* | herb | height [cm] | 14 | **2.61** | **0.008** | **40.2** |
| *Vicia sepium* | herb | leaf area [mm^2^] | 14 | 0.87 | 0.38 | 12.2 |
| *Vicia sepium* | herb | SLA [mm^2^/mg] | 14 | **1.91** | **0.05** | **23.3** |
| *Vicia sepium* | herb | LDMC [mg/g] | 14 | **-5.04** | **<0.001** | **-19.8** |

* the relative change in % of the mean response

**Figure S3** Values of height, leaf area, specific leaf area (SLA), and leaf dry matter content (LDMC) for plots dominated by *Lupinus polyphyllus* (inv. red) and those without invasive species (cont. blue) for grass species. The line represents the median, the box the interquartile range, the whiskers the range of non-outlying values, and small dots indicate outliers.


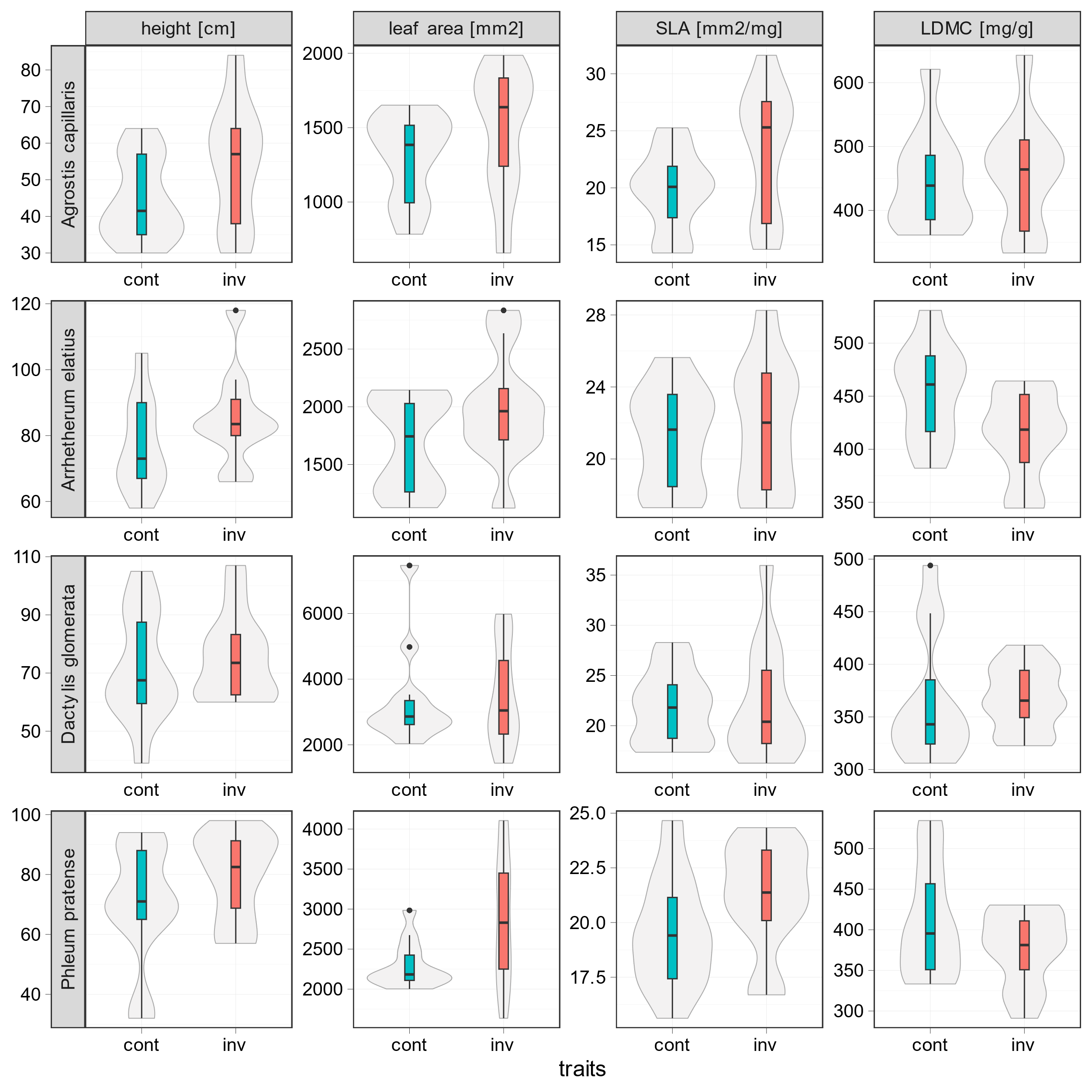


**Figure S4** Values of height, leaf area, specific leaf area (SLA), and leaf dry matter content (LDMC) for plots dominated by *Lupinus polyphyllus* (inv. red) and those without invasive species (cont. blue) for herbs. The line represents the median, the box the interquartile range, the whiskers the range of non-outlying values, and small dots indicate outliers.


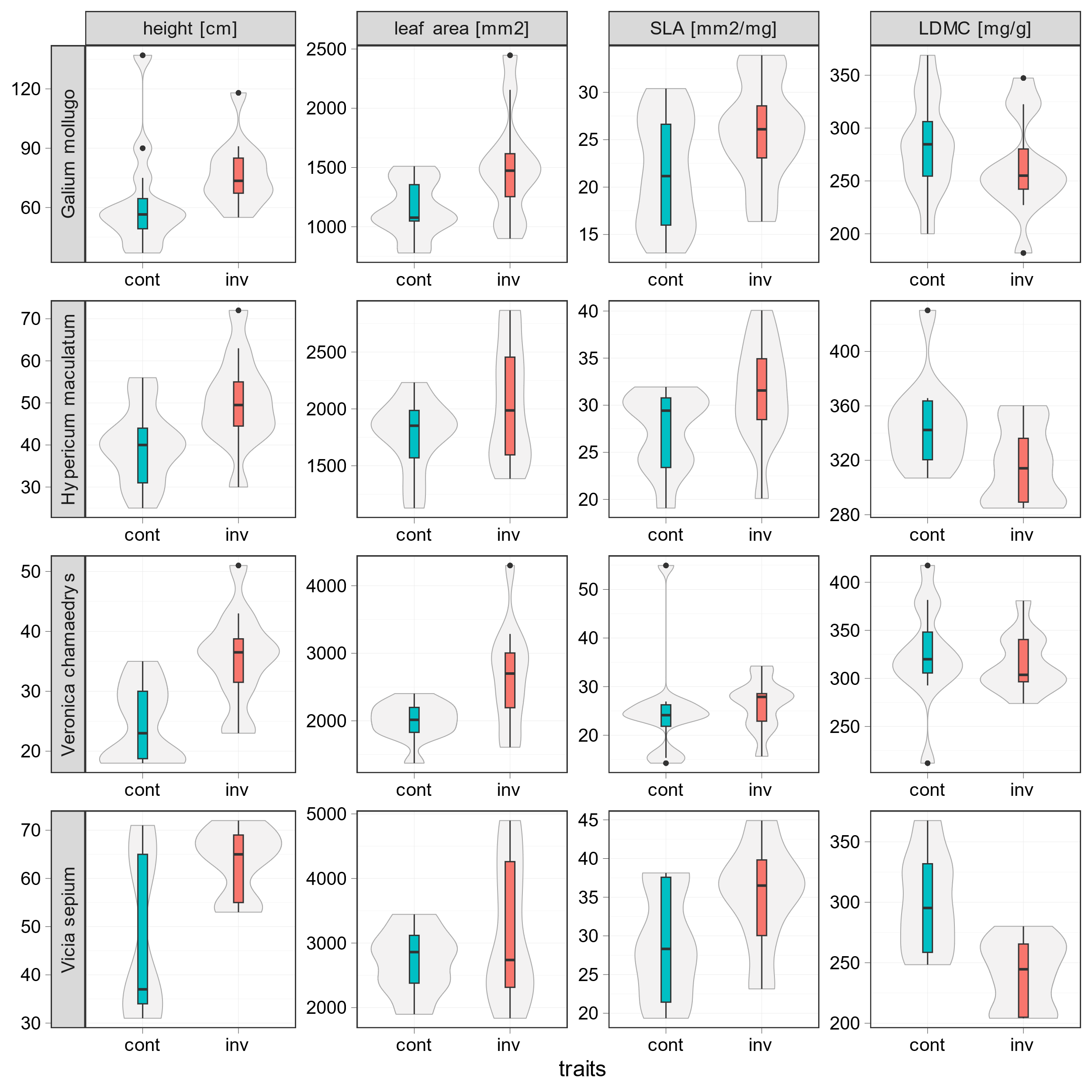


**Figure S5.** Values of species traits for herbs versus environmental variables for plots dominated by *Lupinus polyphyllus* (inv. red) and those without invasive species (cont. blue).


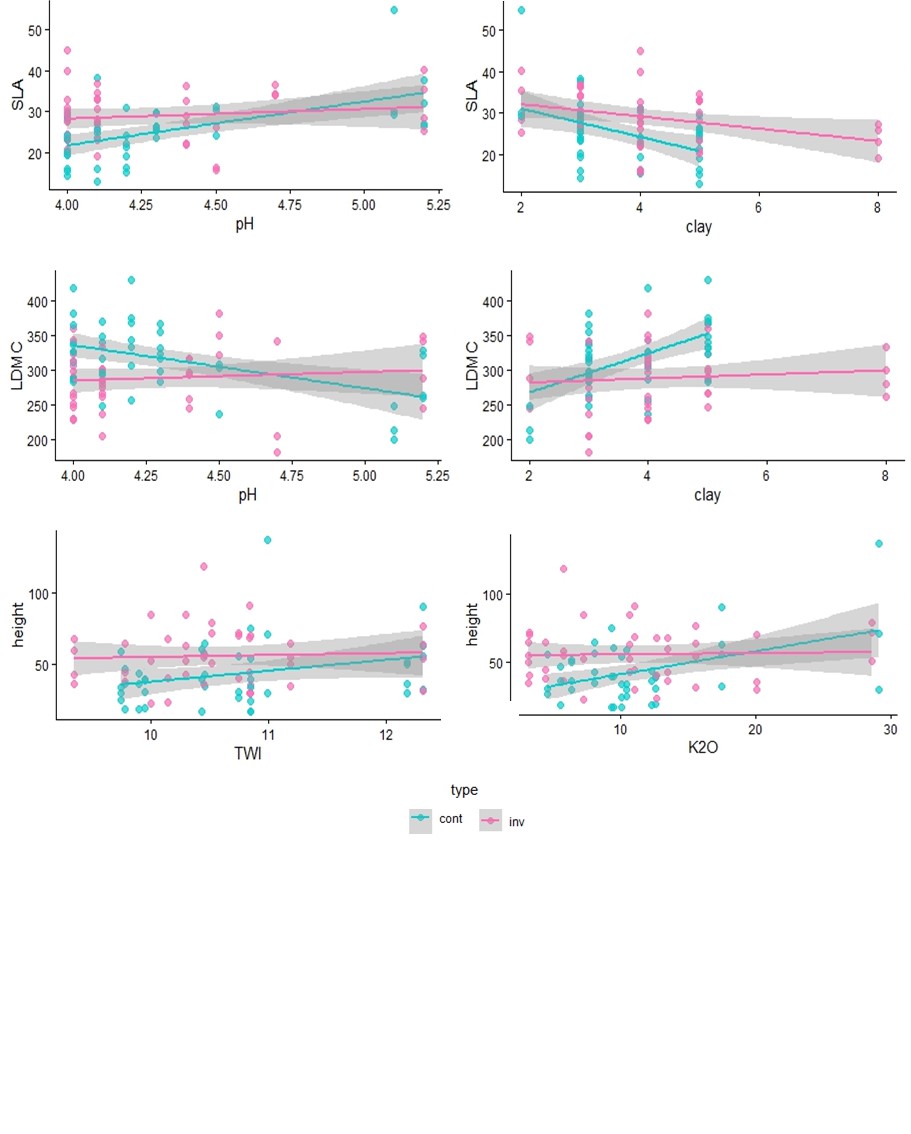


**Figure S6.** Values of species traits for grasses versus environmental variables for plots dominated by *Lupinus polyphyllus* (inv. red) and those without invasive species (cont. blue).


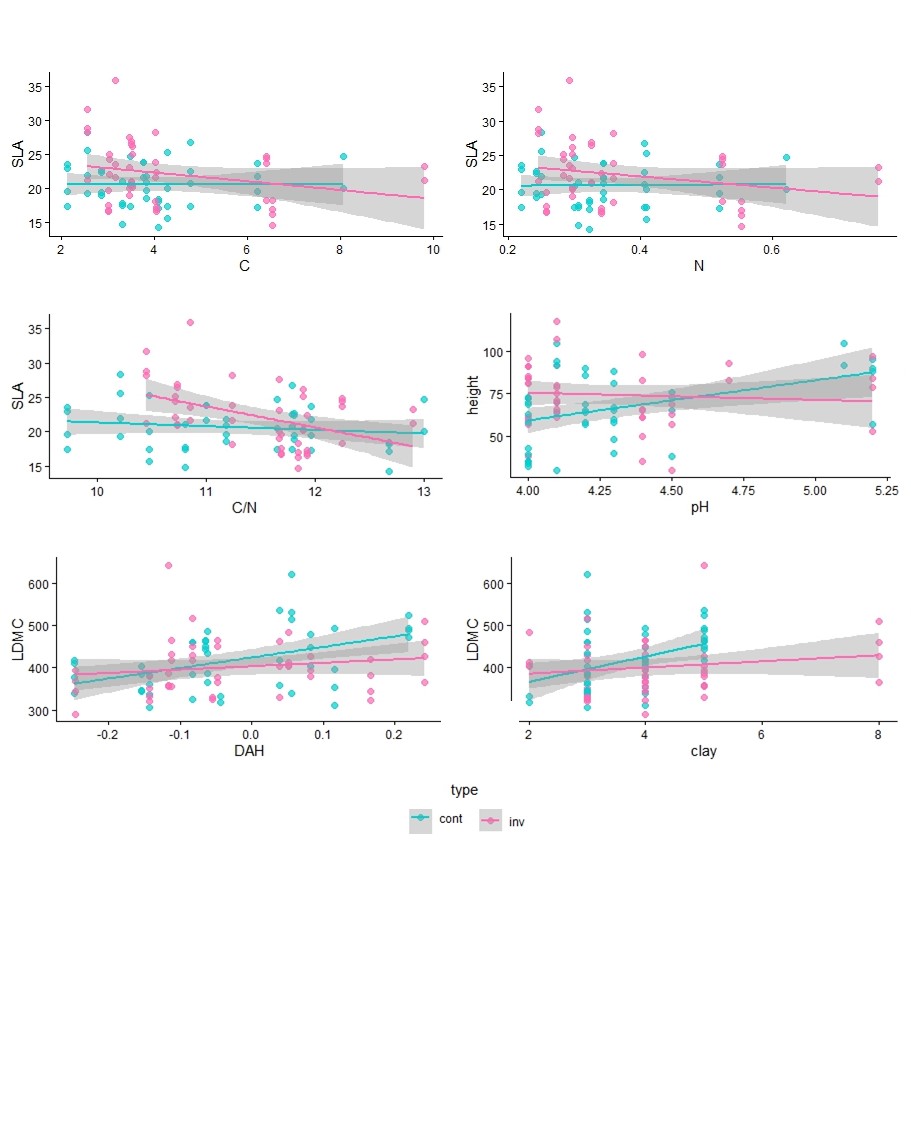


**Table S4.** Median values and changes (delta) in coordinates along the CSR triangle axes (strategy) for target species (species) in invaded and control plots, with corresponding statistical test results (Z, p). Bolding letters indicate significant differences.

| **species** | **plant type** | **strategy** | **N** | **Z** | **p** | **invaded** | **control** | **delta*** |
| --- | --- | --- | --- | --- | --- | --- | --- | --- |
| *Agrostis capillaris* | grass | C | 20 | 0.08 | 0.936 | 20.834 | 19.878 | 0.956 |
| *Agrostis capillaris* | grass | S | 20 | -1.08 | 0.28 | 56.525 | 58.884 | -2.359 |
| *Agrostis capillaris* | grass | R | 20 | 1.09 | 0.275 | 23.455 | 20.567 | 2.888 |
| *Arrhetherum elatius* | grass | C | 20 | 2.80 | **0.005** | 24.347 | 20.83 | 3.517 |
| *Arrhetherum elatius* | grass | S | 20 | -4.29 | **<0.001** | 51.602 | 58.09 | -6.488 |
| *Arrhetherum elatius* | grass | R | 20 | 1.54 | 0.122 | 22.012 | 21.004 | 1.008 |
| *Dactylis glomerata* | grass | C | 24 | -0.18 | 0.86 | 32.235 | 30.687 | 1.548 |
| *Dactylis glomerata* | grass | S | 24 | 0.22 | 0.824 | 45.5 | 44.885 | 0.615 |
| *Dactylis glomerata* | grass | R | 24 | -0.40 | 0.692 | 19.37 | 21.216 | -1.846 |
| *Galium mollugo* | herb | C | 24 | 1.99 | **0.05** | 29.786 | 25.703 | 4.083 |
| *Galium mollugo* | herb | S | 24 | -2.49 | **0.01** | 33.652 | 42.38 | -8.728 |
| *Galium mollugo* | herb | R | 24 | 1.60 | 0.109 | 36.774 | 30.037 | 6.737 |
| *Hypericum maculatum* | herb | C | 22 | 8.82 | **0.003** | 26.946 | 25.128 | 1.818 |
| *Hypericum maculatum* | herb | S | 22 | -3.98 | **<0.001** | 38.706 | 45.802 | -7.096 |
| *Hypericum maculatum* | herb | R | 22 | 1.91 | **0.05** | 35.021 | 30.461 | 4.56 |
| *Phleum pratense* | grass | C | 24 | 2.44 | **0.01** | 29.612 | 27.205 | 2.407 |
| *Phleum pratense* | grass | S | 24 | -2.64 | **0.008** | 47.081 | 51.662 | -4.581 |
| *Phleum pratense* | grass | R | 24 | 1.49 | 0.135 | 21.299 | 18.993 | 2.306 |
| *Veronica chamaedrys* | herb | C | 24 | 3.08 | **0.002** | 30.871 | 27.971 | 2.9 |
| *Veronica chamaedrys* | herb | S | 24 | -0.98 | 0.327 | 38.984 | 42.879 | -3.895 |
| *Veronica chamaedrys* | herb | R | 24 | 0.723 | 0.469 | 29.838 | 28.567 | 1.271 |
| *Vicia sepium* | herb | C | 14 | 4.48 | **<0.001** | 37.284 | 32.108 | 5.176 |
| *Vicia sepium* | herb | S | 14 | -4.563 | **<0.001** | 24.406 | 34.191 | -9.785 |
| *Vicia sepium* | herb | R | 14 | 3.21 | **0.001** | 40.509 | 32.371 | 7.799 |

*delta = median_inv - median_noninv

**Figure S7.** Location of average values for species and plots within the CRS strategy triangle for grass species along with PERMANOVA results (F and p) comparing CSR strategy differences between plants in invaded (red points) and control (blue points) plots.


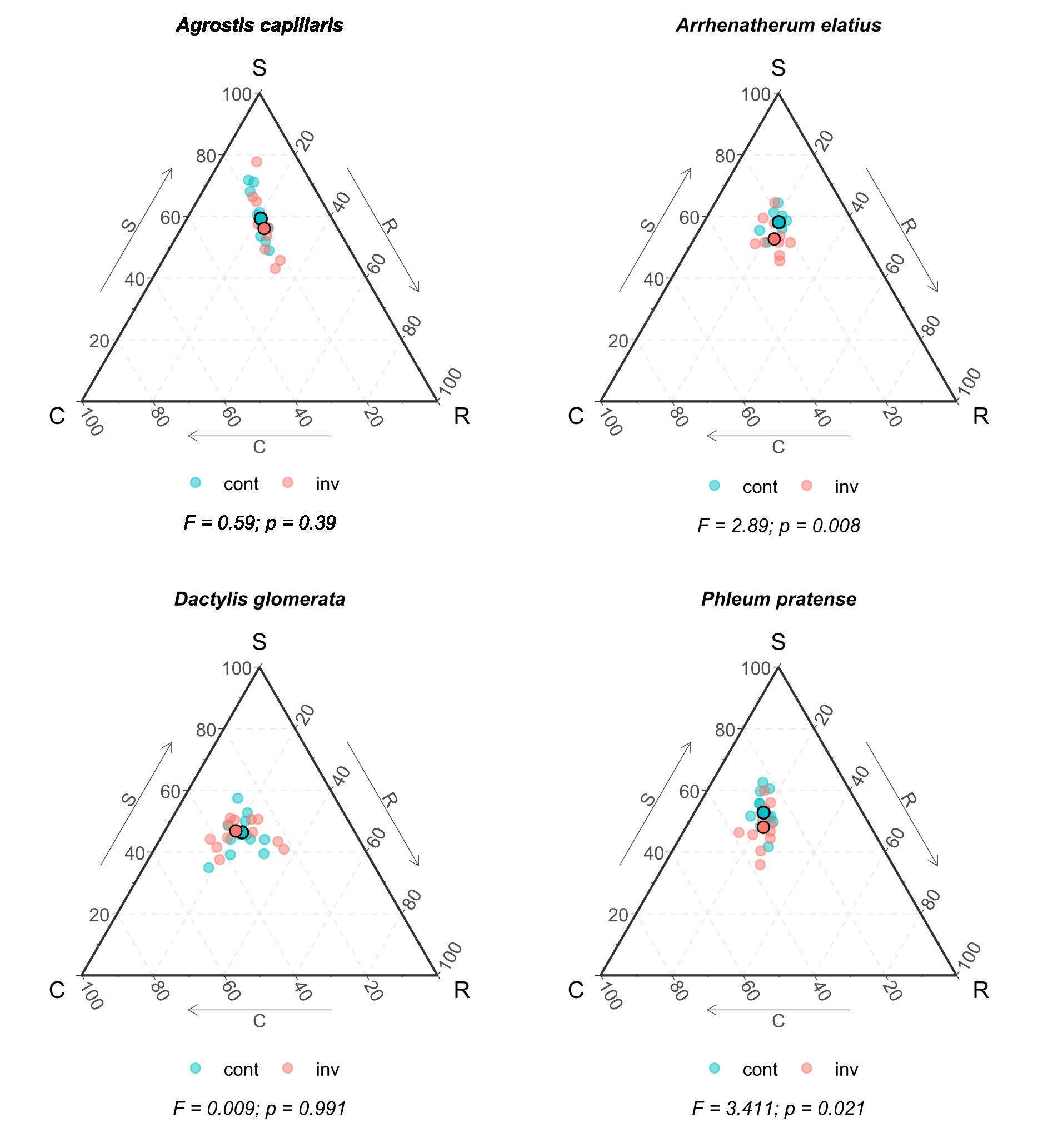


**Figure S8.** Location of average values for species and plots within the CRS strategy triangle for herbs along with PERMANOVA results (F and p) comparing CSR strategy differences between plants in invaded (red points) and control (blue points) plots.


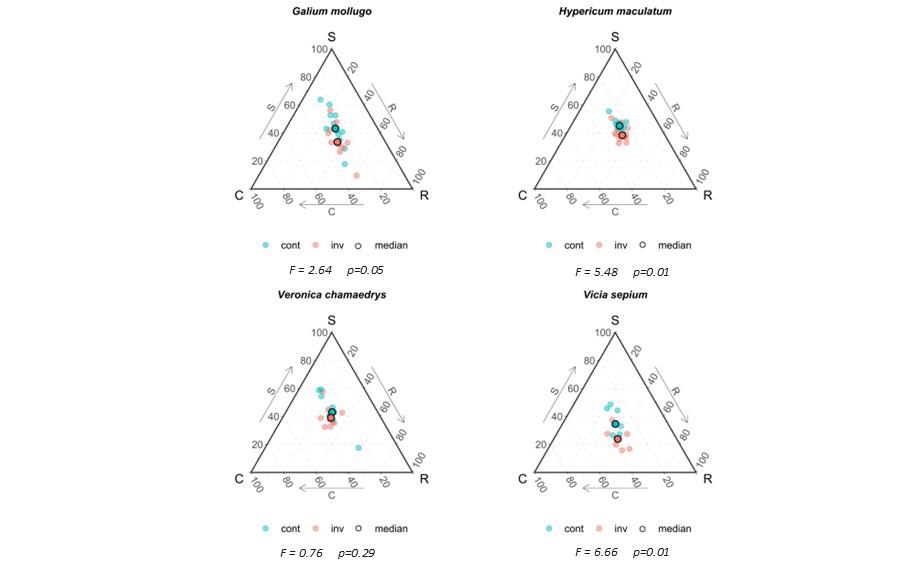


**Table S5.** Spearman rank correlation matrix (r - upper triangle. p - lower triangel. ) among median of species height and effect size (ef) for height (ef-height). leaf dry matter content (ef_LDMC). leaf area (ef_LA) and specific leaf area (ef_SLA).

|  | height | ef_height | ef_LDMC | ef_LA | ef_SLA |
| --- | --- | --- | --- | --- | --- |
| height | - | -8.857 | -0.048 | -0.312 | -0.524 |
| ef_height | 0.011 | - | -0.309 | 0.503 | 0.690 |
| ef_LDMC | 0.935 | 0.462 | - | -0.156 | -0.286 |
| ef_LA | 0.449 | 0.208 | 0.713 | - | 0.132 |
| ef_SLA | 0.196 | 0.069 | 0.501 | 0757 | - |

**Figure S9.** Landscape of mountain grasslands invaded by *Lupinus polyphyllus* (upper panel), and adjacent grasslands without invasive species (bottom panel) (photos by Boglárka Berki, 25.06.2024).


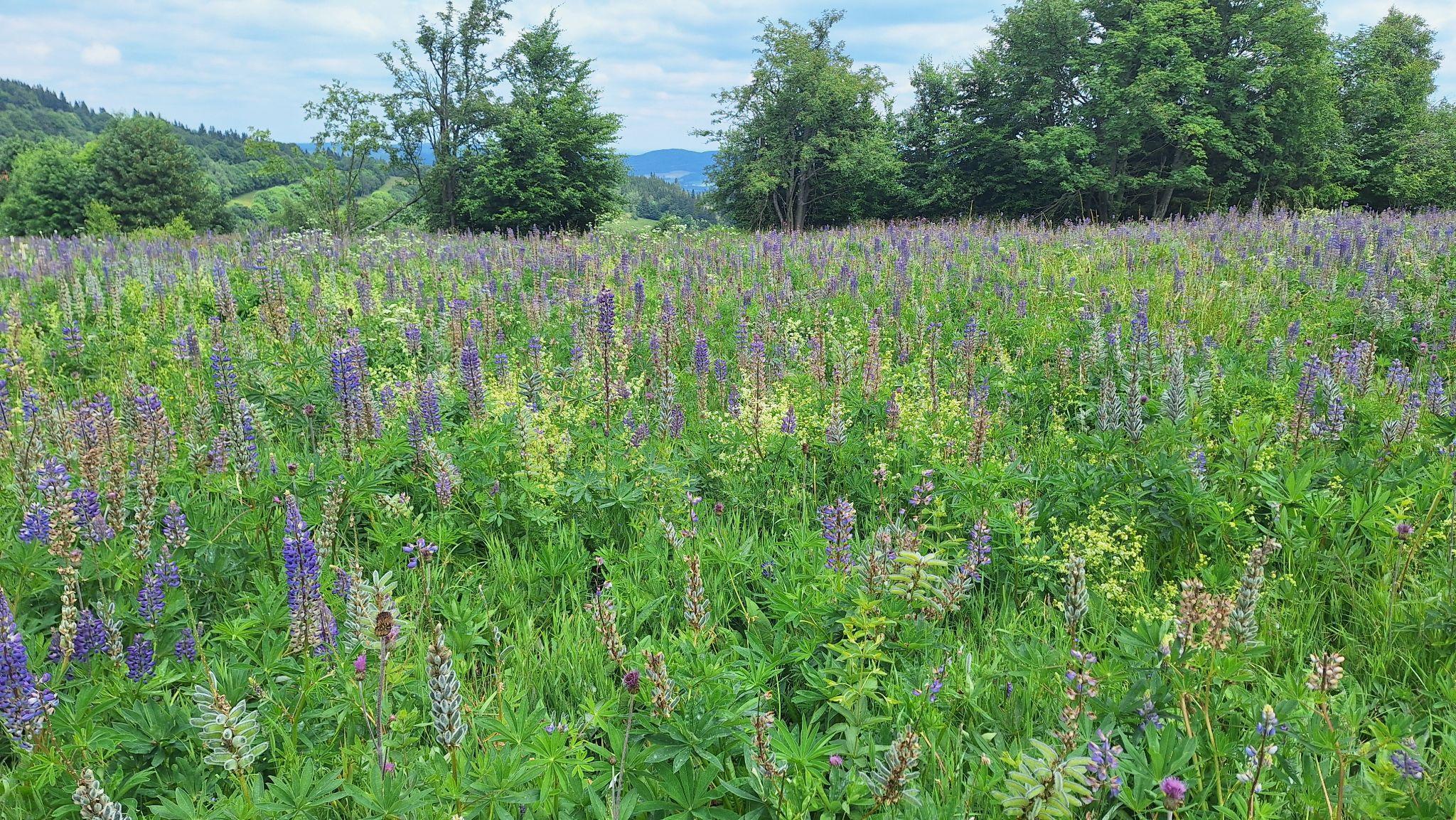


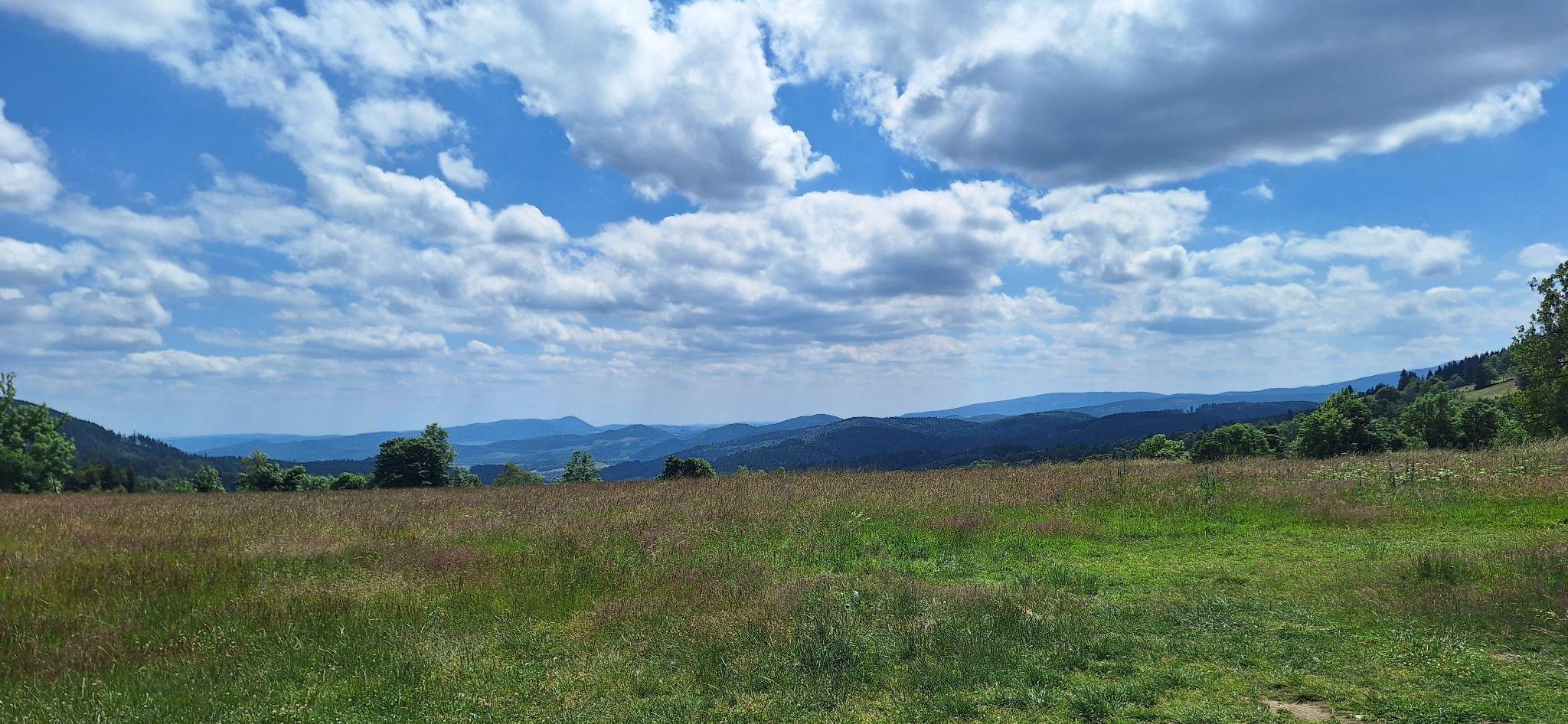


**Figure S10.** Comparison of the native plants' size grown in grasslands invaded by *Lupinus polyphyllus* (left plant) and without invasive species (right plant) (photos by Marta Czarniecka-Wiera, 25.06.2024).

| 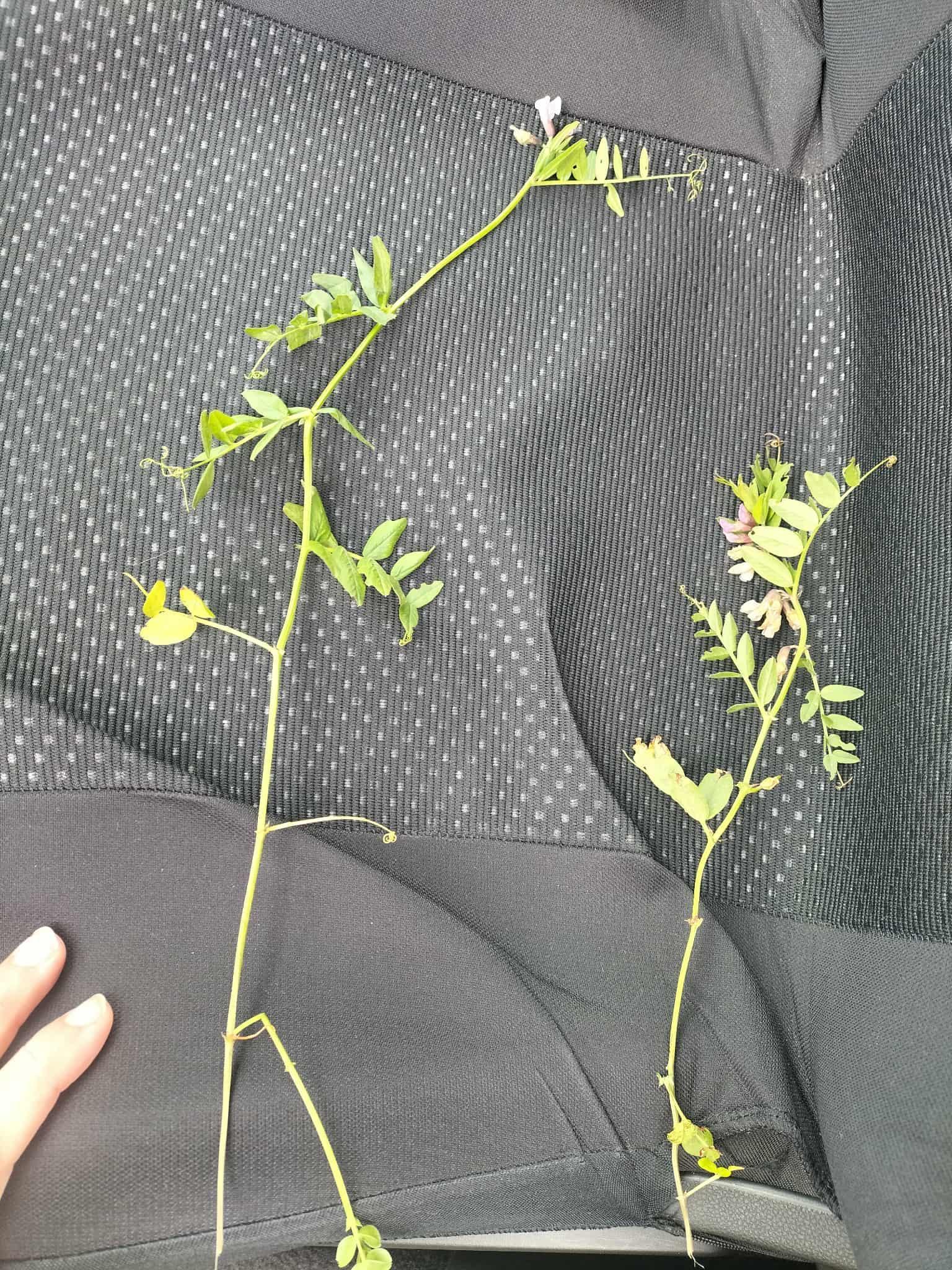 | 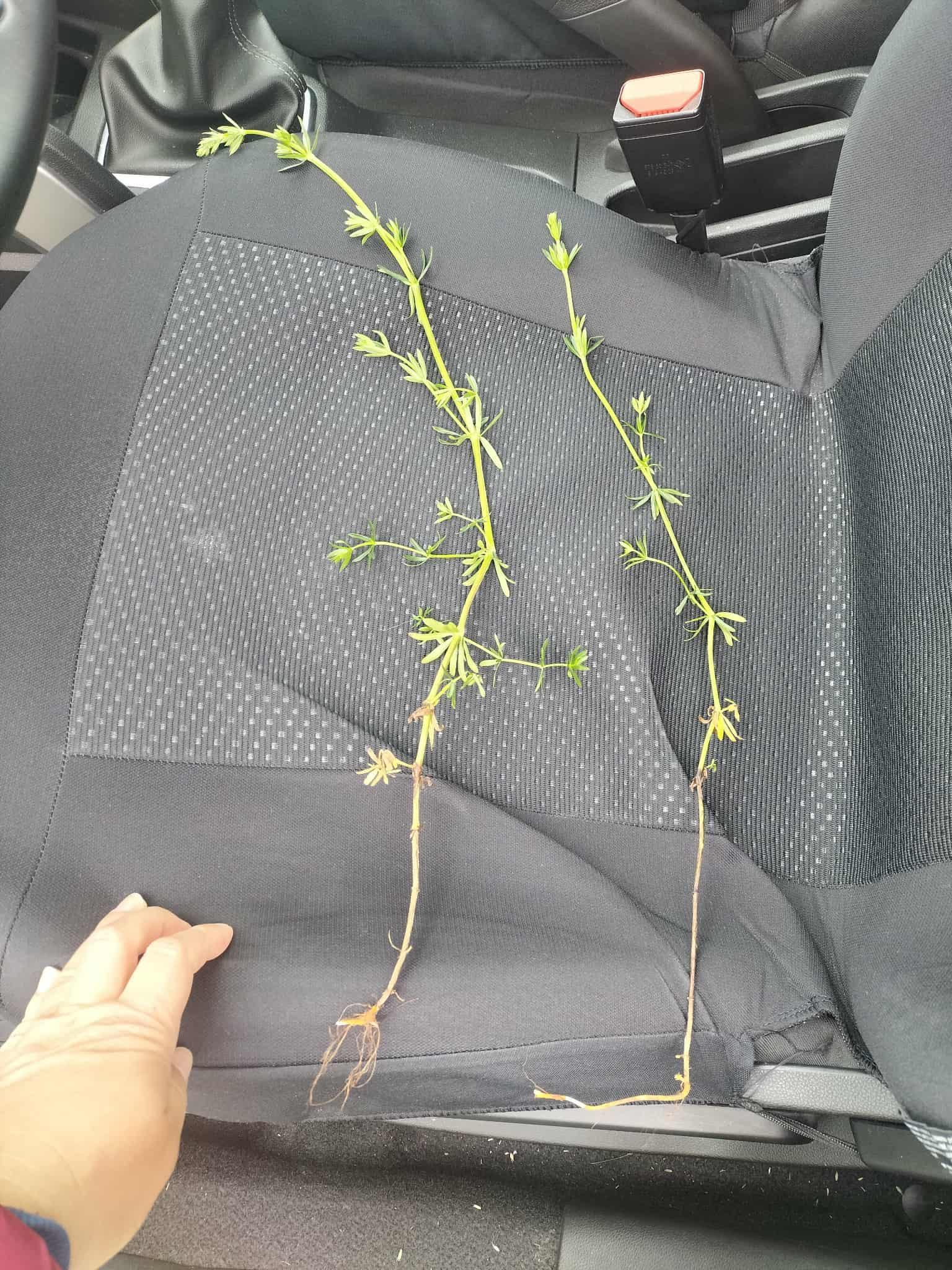 |
| --- | --- |
| *Vicia sepium* | *Galium mollugo* |
| 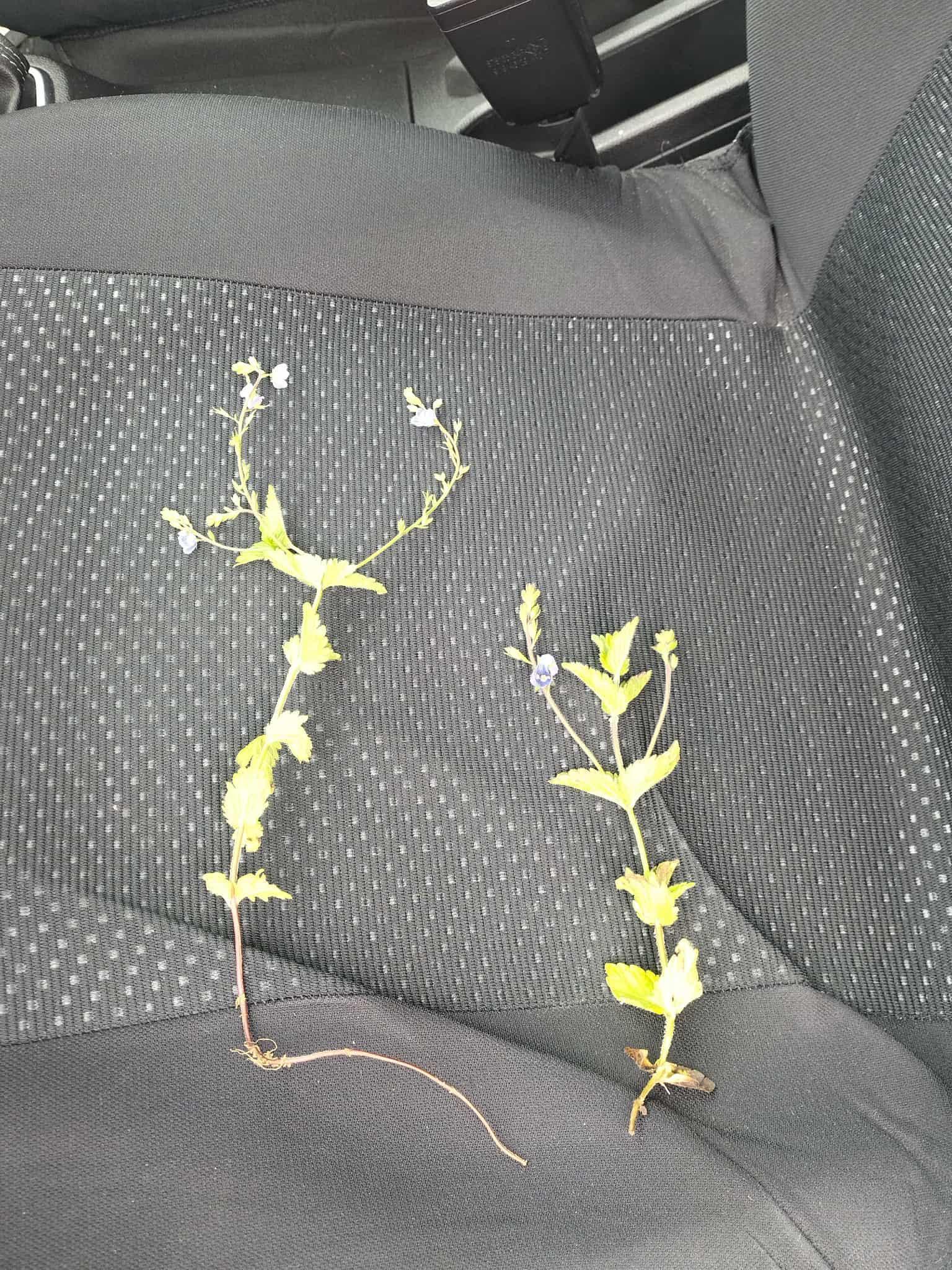 | |
| *Veronica chamaedrys* | |
