# Supplementary figures and images for: Invasive Lupinus polyphyllus Alters Functional Traits and Life Strategies of Native Species
Source: Ecol Evol. 2026 Jun 29;16(7):e73911. doi: 10.1002/ece3.73911 (PMC13314384; doi:10.1002/ece3.73911)

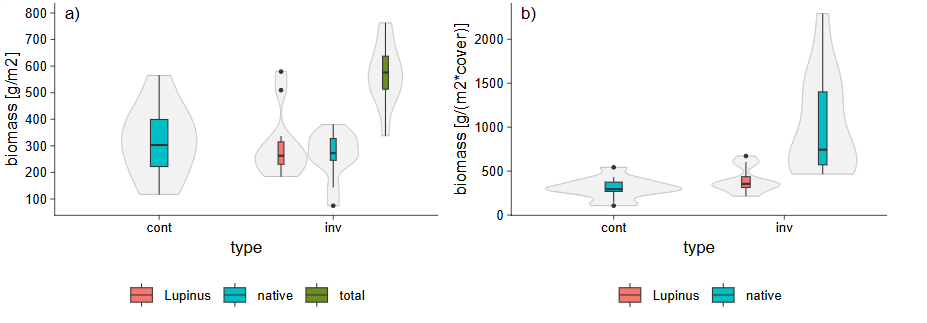

Supplement: Supplementary file 2 — Data S2: ece373911‐sup‐0002‐Supinfo.zip. Table S1: Geographical coordinates, spatial distance between paired plots at a site, species richness, aboveground biomass, and values of environmental variables for paired plots with results of statistical tests of differences between invaded and control plots (Chi2, p and distribution family). The abbreviations of variable names: N species—species richness, biomass—aboveground biomass, alt—altitude, TWI—topographic wetness index, DAH—diurnal anisotropic heating, N‐total nitrogen, C—total carbon, P—available phosphorus (P2O5), K—available potassium (K2O), Mg—available magnesium, pH—soil pH measured in a 1 M KCl solution, coarse—coarse soil fraction (particle diameter above 2 mm), sand—sand fraction (2–0.05 mm), silt—silt fraction (0.05–0.002 mm), and clay—clay fraction (< 0.002 mm in diameter). Multivariate PCA analysis for comparison of soil parameters and topographic factors between control and invaded plots. Table S2: Values of loadings and explained variance in PCA analysis of environmental variables. The loadings with highest value in particular PCA axis are bolded. Variable names abbreviation the same as in Table S2. Table S3: Results of statistical tests (Z, p, effect size) for functional traits between plots invaded by Lupinus polyphyllus and control plots for particular species (species). The significant differences are highlighted in bold. Additionally shown is the affinity of a species to plant functional types (plant type), number of observed pairs (N), as well as effect size. Table S4: Median values and changes (delta) in coordinates along the CSR triangle axes (strategy) for target species (species) in invaded and control plots with corresponding statistical test results (Z, p). Bolding letters indicate significant differences. Table S5: Spearman rank correlation matrix (r—upper triangle, p—lower triangle) among median of species height and effect size (ef) for height (ef‐height), leaf dry matter content (ef_LD [file ECE3-16-e73911-s001.zip › FigS2.tiff]

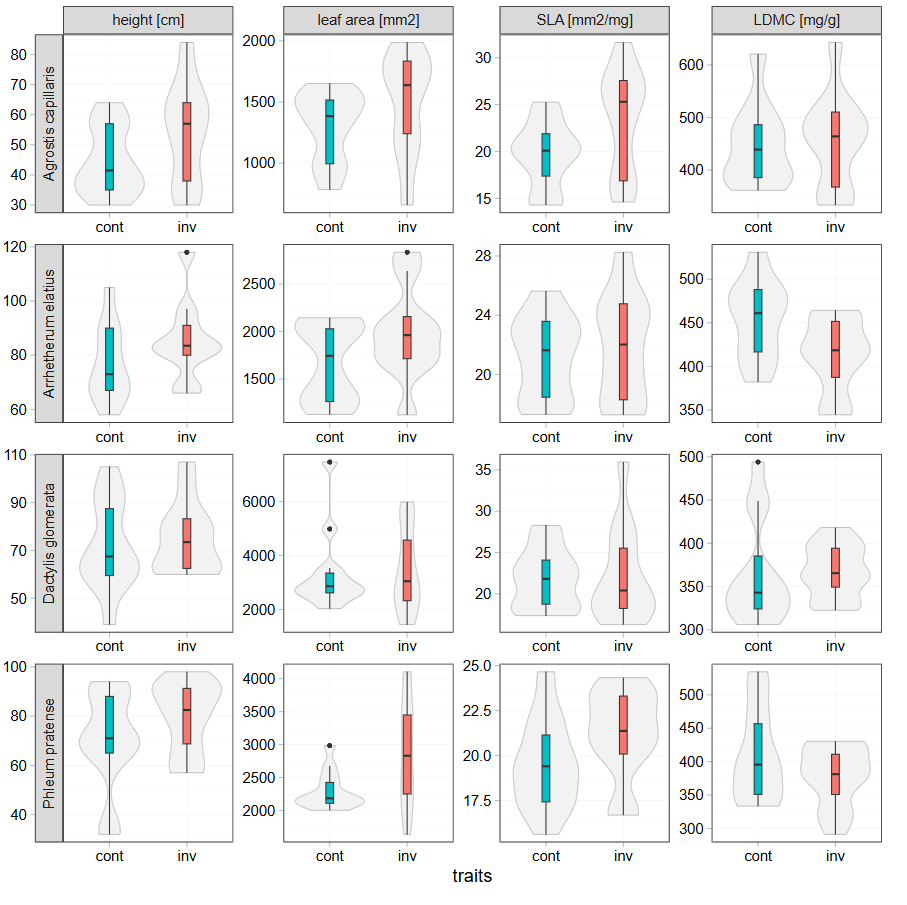

Supplement: Supplementary file 2 — Data S2: ece373911‐sup‐0002‐Supinfo.zip. Table S1: Geographical coordinates, spatial distance between paired plots at a site, species richness, aboveground biomass, and values of environmental variables for paired plots with results of statistical tests of differences between invaded and control plots (Chi2, p and distribution family). The abbreviations of variable names: N species—species richness, biomass—aboveground biomass, alt—altitude, TWI—topographic wetness index, DAH—diurnal anisotropic heating, N‐total nitrogen, C—total carbon, P—available phosphorus (P2O5), K—available potassium (K2O), Mg—available magnesium, pH—soil pH measured in a 1 M KCl solution, coarse—coarse soil fraction (particle diameter above 2 mm), sand—sand fraction (2–0.05 mm), silt—silt fraction (0.05–0.002 mm), and clay—clay fraction (< 0.002 mm in diameter). Multivariate PCA analysis for comparison of soil parameters and topographic factors between control and invaded plots. Table S2: Values of loadings and explained variance in PCA analysis of environmental variables. The loadings with highest value in particular PCA axis are bolded. Variable names abbreviation the same as in Table S2. Table S3: Results of statistical tests (Z, p, effect size) for functional traits between plots invaded by Lupinus polyphyllus and control plots for particular species (species). The significant differences are highlighted in bold. Additionally shown is the affinity of a species to plant functional types (plant type), number of observed pairs (N), as well as effect size. Table S4: Median values and changes (delta) in coordinates along the CSR triangle axes (strategy) for target species (species) in invaded and control plots with corresponding statistical test results (Z, p). Bolding letters indicate significant differences. Table S5: Spearman rank correlation matrix (r—upper triangle, p—lower triangle) among median of species height and effect size (ef) for height (ef‐height), leaf dry matter content (ef_LD [file ECE3-16-e73911-s001.zip › FigS3.tiff]

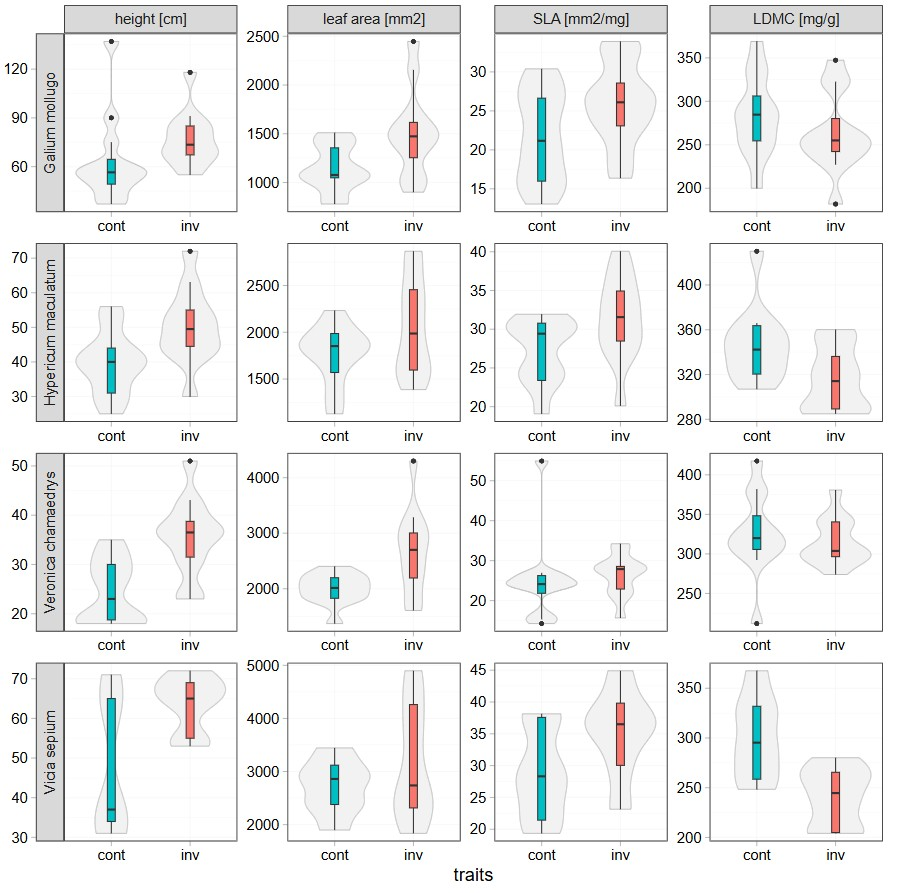

Supplement: Supplementary file 2 — Data S2: ece373911‐sup‐0002‐Supinfo.zip. Table S1: Geographical coordinates, spatial distance between paired plots at a site, species richness, aboveground biomass, and values of environmental variables for paired plots with results of statistical tests of differences between invaded and control plots (Chi2, p and distribution family). The abbreviations of variable names: N species—species richness, biomass—aboveground biomass, alt—altitude, TWI—topographic wetness index, DAH—diurnal anisotropic heating, N‐total nitrogen, C—total carbon, P—available phosphorus (P2O5), K—available potassium (K2O), Mg—available magnesium, pH—soil pH measured in a 1 M KCl solution, coarse—coarse soil fraction (particle diameter above 2 mm), sand—sand fraction (2–0.05 mm), silt—silt fraction (0.05–0.002 mm), and clay—clay fraction (< 0.002 mm in diameter). Multivariate PCA analysis for comparison of soil parameters and topographic factors between control and invaded plots. Table S2: Values of loadings and explained variance in PCA analysis of environmental variables. The loadings with highest value in particular PCA axis are bolded. Variable names abbreviation the same as in Table S2. Table S3: Results of statistical tests (Z, p, effect size) for functional traits between plots invaded by Lupinus polyphyllus and control plots for particular species (species). The significant differences are highlighted in bold. Additionally shown is the affinity of a species to plant functional types (plant type), number of observed pairs (N), as well as effect size. Table S4: Median values and changes (delta) in coordinates along the CSR triangle axes (strategy) for target species (species) in invaded and control plots with corresponding statistical test results (Z, p). Bolding letters indicate significant differences. Table S5: Spearman rank correlation matrix (r—upper triangle, p—lower triangle) among median of species height and effect size (ef) for height (ef‐height), leaf dry matter content (ef_LD [file ECE3-16-e73911-s001.zip › FigS4.tiff]

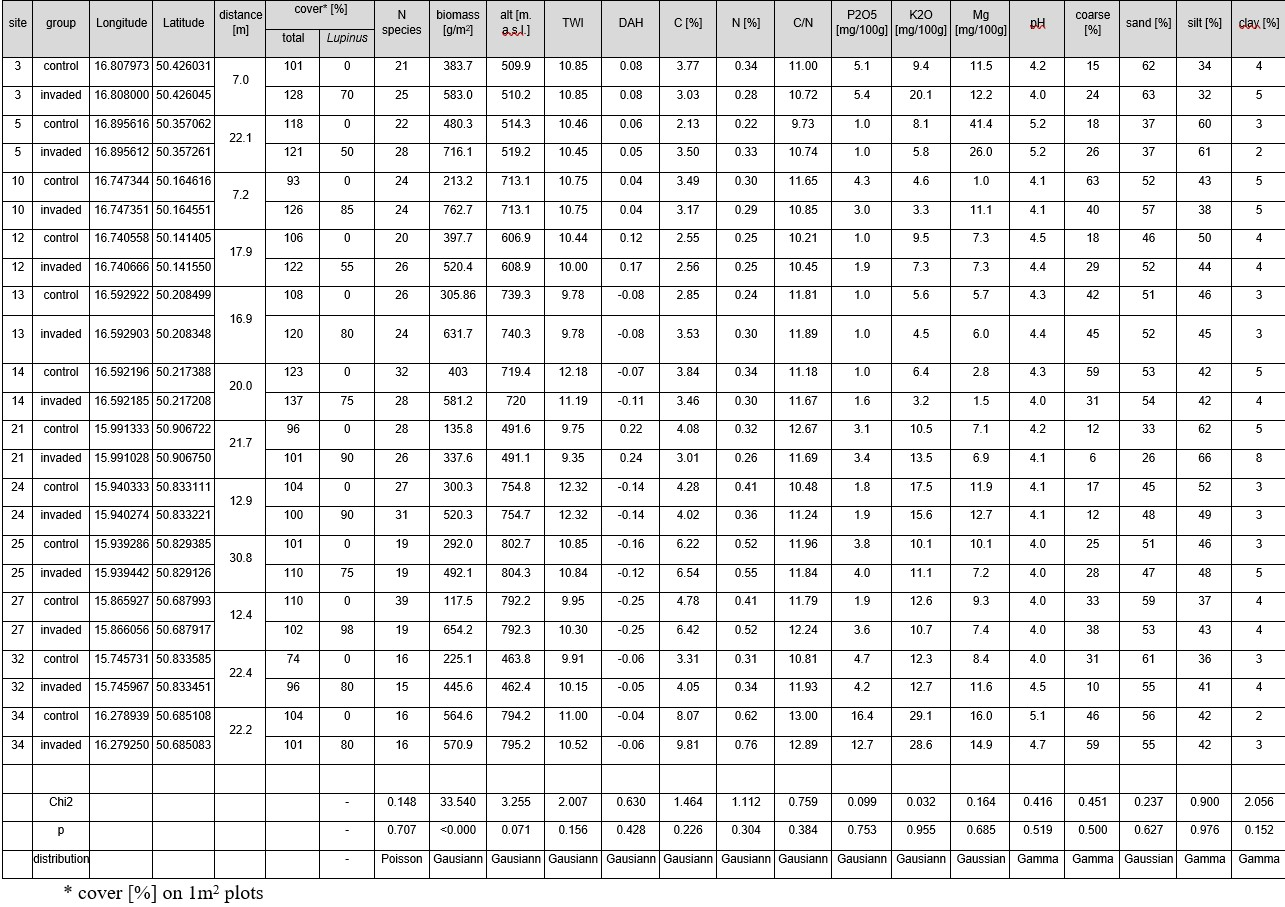

Supplement: Supplementary file 2 — Data S2: ece373911‐sup‐0002‐Supinfo.zip. Table S1: Geographical coordinates, spatial distance between paired plots at a site, species richness, aboveground biomass, and values of environmental variables for paired plots with results of statistical tests of differences between invaded and control plots (Chi2, p and distribution family). The abbreviations of variable names: N species—species richness, biomass—aboveground biomass, alt—altitude, TWI—topographic wetness index, DAH—diurnal anisotropic heating, N‐total nitrogen, C—total carbon, P—available phosphorus (P2O5), K—available potassium (K2O), Mg—available magnesium, pH—soil pH measured in a 1 M KCl solution, coarse—coarse soil fraction (particle diameter above 2 mm), sand—sand fraction (2–0.05 mm), silt—silt fraction (0.05–0.002 mm), and clay—clay fraction (< 0.002 mm in diameter). Multivariate PCA analysis for comparison of soil parameters and topographic factors between control and invaded plots. Table S2: Values of loadings and explained variance in PCA analysis of environmental variables. The loadings with highest value in particular PCA axis are bolded. Variable names abbreviation the same as in Table S2. Table S3: Results of statistical tests (Z, p, effect size) for functional traits between plots invaded by Lupinus polyphyllus and control plots for particular species (species). The significant differences are highlighted in bold. Additionally shown is the affinity of a species to plant functional types (plant type), number of observed pairs (N), as well as effect size. Table S4: Median values and changes (delta) in coordinates along the CSR triangle axes (strategy) for target species (species) in invaded and control plots with corresponding statistical test results (Z, p). Bolding letters indicate significant differences. Table S5: Spearman rank correlation matrix (r—upper triangle, p—lower triangle) among median of species height and effect size (ef) for height (ef‐height), leaf dry matter content (ef_LD [file ECE3-16-e73911-s001.zip › TableS1.tiff]

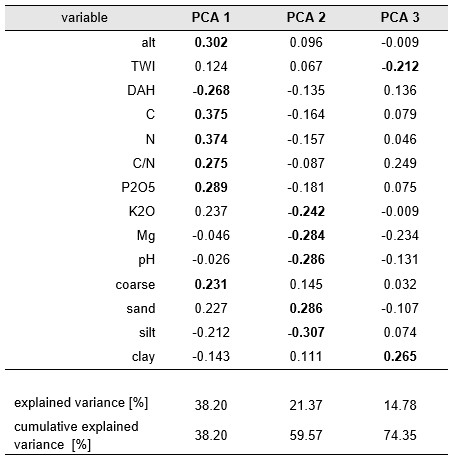

Supplement: Supplementary file 2 — Data S2: ece373911‐sup‐0002‐Supinfo.zip. Table S1: Geographical coordinates, spatial distance between paired plots at a site, species richness, aboveground biomass, and values of environmental variables for paired plots with results of statistical tests of differences between invaded and control plots (Chi2, p and distribution family). The abbreviations of variable names: N species—species richness, biomass—aboveground biomass, alt—altitude, TWI—topographic wetness index, DAH—diurnal anisotropic heating, N‐total nitrogen, C—total carbon, P—available phosphorus (P2O5), K—available potassium (K2O), Mg—available magnesium, pH—soil pH measured in a 1 M KCl solution, coarse—coarse soil fraction (particle diameter above 2 mm), sand—sand fraction (2–0.05 mm), silt—silt fraction (0.05–0.002 mm), and clay—clay fraction (< 0.002 mm in diameter). Multivariate PCA analysis for comparison of soil parameters and topographic factors between control and invaded plots. Table S2: Values of loadings and explained variance in PCA analysis of environmental variables. The loadings with highest value in particular PCA axis are bolded. Variable names abbreviation the same as in Table S2. Table S3: Results of statistical tests (Z, p, effect size) for functional traits between plots invaded by Lupinus polyphyllus and control plots for particular species (species). The significant differences are highlighted in bold. Additionally shown is the affinity of a species to plant functional types (plant type), number of observed pairs (N), as well as effect size. Table S4: Median values and changes (delta) in coordinates along the CSR triangle axes (strategy) for target species (species) in invaded and control plots with corresponding statistical test results (Z, p). Bolding letters indicate significant differences. Table S5: Spearman rank correlation matrix (r—upper triangle, p—lower triangle) among median of species height and effect size (ef) for height (ef‐height), leaf dry matter content (ef_LD [file ECE3-16-e73911-s001.zip › TableS2.tiff]

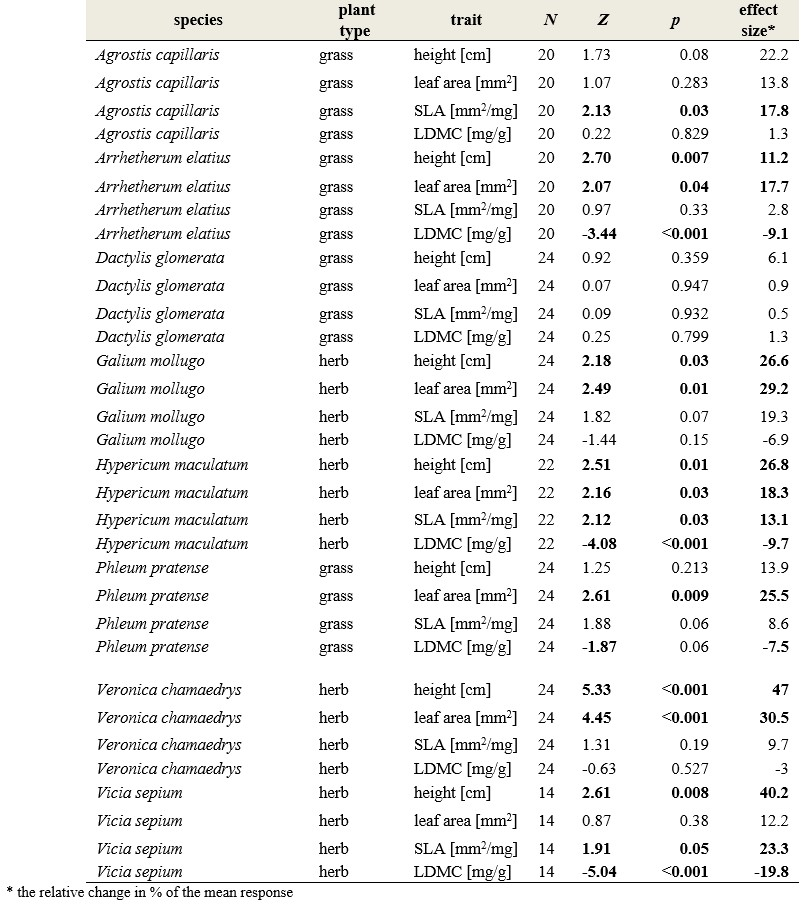

Supplement: Supplementary file 2 — Data S2: ece373911‐sup‐0002‐Supinfo.zip. Table S1: Geographical coordinates, spatial distance between paired plots at a site, species richness, aboveground biomass, and values of environmental variables for paired plots with results of statistical tests of differences between invaded and control plots (Chi2, p and distribution family). The abbreviations of variable names: N species—species richness, biomass—aboveground biomass, alt—altitude, TWI—topographic wetness index, DAH—diurnal anisotropic heating, N‐total nitrogen, C—total carbon, P—available phosphorus (P2O5), K—available potassium (K2O), Mg—available magnesium, pH—soil pH measured in a 1 M KCl solution, coarse—coarse soil fraction (particle diameter above 2 mm), sand—sand fraction (2–0.05 mm), silt—silt fraction (0.05–0.002 mm), and clay—clay fraction (< 0.002 mm in diameter). Multivariate PCA analysis for comparison of soil parameters and topographic factors between control and invaded plots. Table S2: Values of loadings and explained variance in PCA analysis of environmental variables. The loadings with highest value in particular PCA axis are bolded. Variable names abbreviation the same as in Table S2. Table S3: Results of statistical tests (Z, p, effect size) for functional traits between plots invaded by Lupinus polyphyllus and control plots for particular species (species). The significant differences are highlighted in bold. Additionally shown is the affinity of a species to plant functional types (plant type), number of observed pairs (N), as well as effect size. Table S4: Median values and changes (delta) in coordinates along the CSR triangle axes (strategy) for target species (species) in invaded and control plots with corresponding statistical test results (Z, p). Bolding letters indicate significant differences. Table S5: Spearman rank correlation matrix (r—upper triangle, p—lower triangle) among median of species height and effect size (ef) for height (ef‐height), leaf dry matter content (ef_LD [file ECE3-16-e73911-s001.zip › TableS3.tiff]

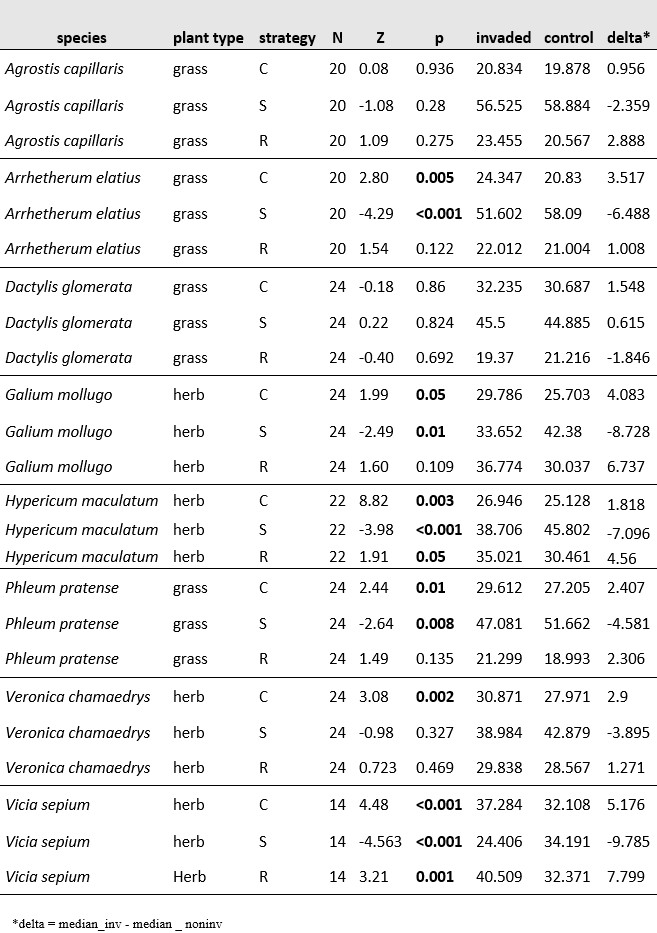

Supplement: Supplementary file 2 — Data S2: ece373911‐sup‐0002‐Supinfo.zip. Table S1: Geographical coordinates, spatial distance between paired plots at a site, species richness, aboveground biomass, and values of environmental variables for paired plots with results of statistical tests of differences between invaded and control plots (Chi2, p and distribution family). The abbreviations of variable names: N species—species richness, biomass—aboveground biomass, alt—altitude, TWI—topographic wetness index, DAH—diurnal anisotropic heating, N‐total nitrogen, C—total carbon, P—available phosphorus (P2O5), K—available potassium (K2O), Mg—available magnesium, pH—soil pH measured in a 1 M KCl solution, coarse—coarse soil fraction (particle diameter above 2 mm), sand—sand fraction (2–0.05 mm), silt—silt fraction (0.05–0.002 mm), and clay—clay fraction (< 0.002 mm in diameter). Multivariate PCA analysis for comparison of soil parameters and topographic factors between control and invaded plots. Table S2: Values of loadings and explained variance in PCA analysis of environmental variables. The loadings with highest value in particular PCA axis are bolded. Variable names abbreviation the same as in Table S2. Table S3: Results of statistical tests (Z, p, effect size) for functional traits between plots invaded by Lupinus polyphyllus and control plots for particular species (species). The significant differences are highlighted in bold. Additionally shown is the affinity of a species to plant functional types (plant type), number of observed pairs (N), as well as effect size. Table S4: Median values and changes (delta) in coordinates along the CSR triangle axes (strategy) for target species (species) in invaded and control plots with corresponding statistical test results (Z, p). Bolding letters indicate significant differences. Table S5: Spearman rank correlation matrix (r—upper triangle, p—lower triangle) among median of species height and effect size (ef) for height (ef‐height), leaf dry matter content (ef_LD [file ECE3-16-e73911-s001.zip › TableS4.tiff]

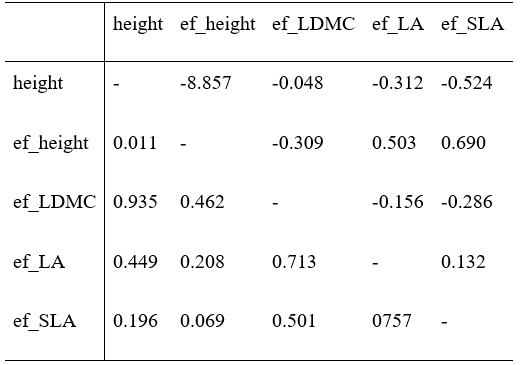

Supplement: Supplementary file 2 — Data S2: ece373911‐sup‐0002‐Supinfo.zip. Table S1: Geographical coordinates, spatial distance between paired plots at a site, species richness, aboveground biomass, and values of environmental variables for paired plots with results of statistical tests of differences between invaded and control plots (Chi2, p and distribution family). The abbreviations of variable names: N species—species richness, biomass—aboveground biomass, alt—altitude, TWI—topographic wetness index, DAH—diurnal anisotropic heating, N‐total nitrogen, C—total carbon, P—available phosphorus (P2O5), K—available potassium (K2O), Mg—available magnesium, pH—soil pH measured in a 1 M KCl solution, coarse—coarse soil fraction (particle diameter above 2 mm), sand—sand fraction (2–0.05 mm), silt—silt fraction (0.05–0.002 mm), and clay—clay fraction (< 0.002 mm in diameter). Multivariate PCA analysis for comparison of soil parameters and topographic factors between control and invaded plots. Table S2: Values of loadings and explained variance in PCA analysis of environmental variables. The loadings with highest value in particular PCA axis are bolded. Variable names abbreviation the same as in Table S2. Table S3: Results of statistical tests (Z, p, effect size) for functional traits between plots invaded by Lupinus polyphyllus and control plots for particular species (species). The significant differences are highlighted in bold. Additionally shown is the affinity of a species to plant functional types (plant type), number of observed pairs (N), as well as effect size. Table S4: Median values and changes (delta) in coordinates along the CSR triangle axes (strategy) for target species (species) in invaded and control plots with corresponding statistical test results (Z, p). Bolding letters indicate significant differences. Table S5: Spearman rank correlation matrix (r—upper triangle, p—lower triangle) among median of species height and effect size (ef) for height (ef‐height), leaf dry matter content (ef_LD [file ECE3-16-e73911-s001.zip › TableS5.tiff]

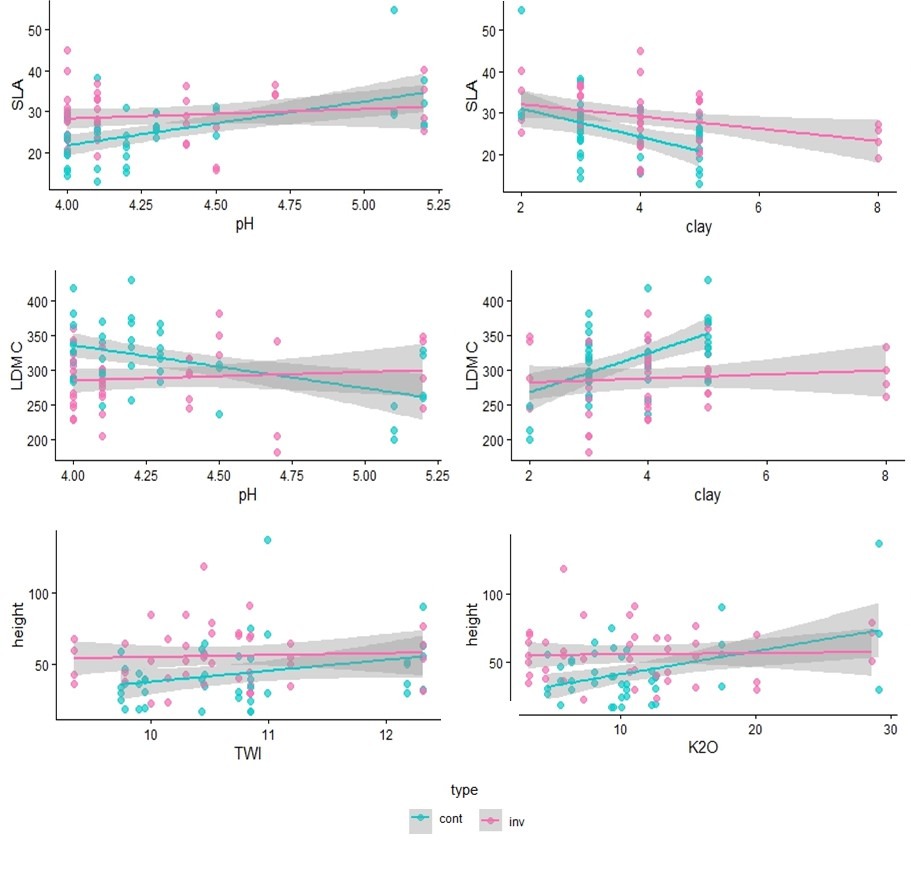

Supplement: Supplementary file 2 — Data S2: ece373911‐sup‐0002‐Supinfo.zip. Table S1: Geographical coordinates, spatial distance between paired plots at a site, species richness, aboveground biomass, and values of environmental variables for paired plots with results of statistical tests of differences between invaded and control plots (Chi2, p and distribution family). The abbreviations of variable names: N species—species richness, biomass—aboveground biomass, alt—altitude, TWI—topographic wetness index, DAH—diurnal anisotropic heating, N‐total nitrogen, C—total carbon, P—available phosphorus (P2O5), K—available potassium (K2O), Mg—available magnesium, pH—soil pH measured in a 1 M KCl solution, coarse—coarse soil fraction (particle diameter above 2 mm), sand—sand fraction (2–0.05 mm), silt—silt fraction (0.05–0.002 mm), and clay—clay fraction (< 0.002 mm in diameter). Multivariate PCA analysis for comparison of soil parameters and topographic factors between control and invaded plots. Table S2: Values of loadings and explained variance in PCA analysis of environmental variables. The loadings with highest value in particular PCA axis are bolded. Variable names abbreviation the same as in Table S2. Table S3: Results of statistical tests (Z, p, effect size) for functional traits between plots invaded by Lupinus polyphyllus and control plots for particular species (species). The significant differences are highlighted in bold. Additionally shown is the affinity of a species to plant functional types (plant type), number of observed pairs (N), as well as effect size. Table S4: Median values and changes (delta) in coordinates along the CSR triangle axes (strategy) for target species (species) in invaded and control plots with corresponding statistical test results (Z, p). Bolding letters indicate significant differences. Table S5: Spearman rank correlation matrix (r—upper triangle, p—lower triangle) among median of species height and effect size (ef) for height (ef‐height), leaf dry matter content (ef_LD [file ECE3-16-e73911-s001.zip › Fig.S5.JPG]

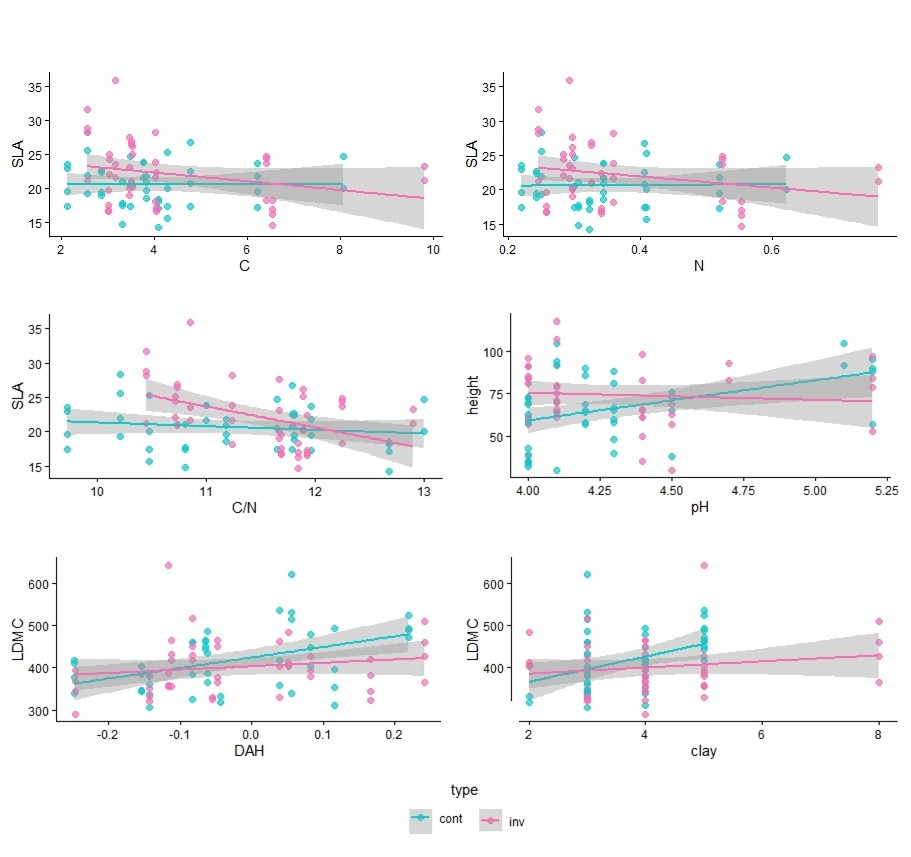

Supplement: Supplementary file 2 — Data S2: ece373911‐sup‐0002‐Supinfo.zip. Table S1: Geographical coordinates, spatial distance between paired plots at a site, species richness, aboveground biomass, and values of environmental variables for paired plots with results of statistical tests of differences between invaded and control plots (Chi2, p and distribution family). The abbreviations of variable names: N species—species richness, biomass—aboveground biomass, alt—altitude, TWI—topographic wetness index, DAH—diurnal anisotropic heating, N‐total nitrogen, C—total carbon, P—available phosphorus (P2O5), K—available potassium (K2O), Mg—available magnesium, pH—soil pH measured in a 1 M KCl solution, coarse—coarse soil fraction (particle diameter above 2 mm), sand—sand fraction (2–0.05 mm), silt—silt fraction (0.05–0.002 mm), and clay—clay fraction (< 0.002 mm in diameter). Multivariate PCA analysis for comparison of soil parameters and topographic factors between control and invaded plots. Table S2: Values of loadings and explained variance in PCA analysis of environmental variables. The loadings with highest value in particular PCA axis are bolded. Variable names abbreviation the same as in Table S2. Table S3: Results of statistical tests (Z, p, effect size) for functional traits between plots invaded by Lupinus polyphyllus and control plots for particular species (species). The significant differences are highlighted in bold. Additionally shown is the affinity of a species to plant functional types (plant type), number of observed pairs (N), as well as effect size. Table S4: Median values and changes (delta) in coordinates along the CSR triangle axes (strategy) for target species (species) in invaded and control plots with corresponding statistical test results (Z, p). Bolding letters indicate significant differences. Table S5: Spearman rank correlation matrix (r—upper triangle, p—lower triangle) among median of species height and effect size (ef) for height (ef‐height), leaf dry matter content (ef_LD [file ECE3-16-e73911-s001.zip › Fig.S6.JPG]

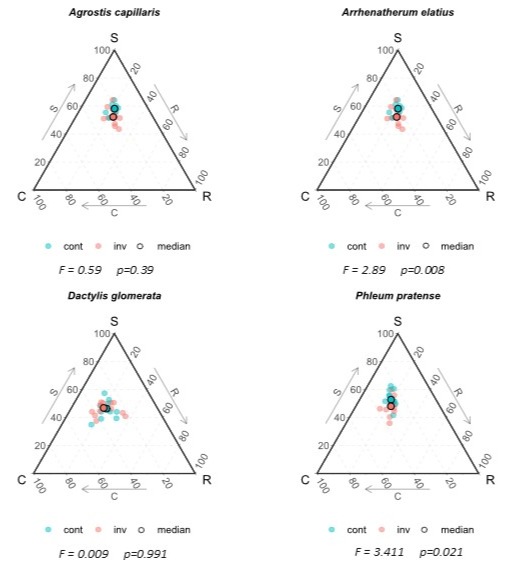

Supplement: Supplementary file 2 — Data S2: ece373911‐sup‐0002‐Supinfo.zip. Table S1: Geographical coordinates, spatial distance between paired plots at a site, species richness, aboveground biomass, and values of environmental variables for paired plots with results of statistical tests of differences between invaded and control plots (Chi2, p and distribution family). The abbreviations of variable names: N species—species richness, biomass—aboveground biomass, alt—altitude, TWI—topographic wetness index, DAH—diurnal anisotropic heating, N‐total nitrogen, C—total carbon, P—available phosphorus (P2O5), K—available potassium (K2O), Mg—available magnesium, pH—soil pH measured in a 1 M KCl solution, coarse—coarse soil fraction (particle diameter above 2 mm), sand—sand fraction (2–0.05 mm), silt—silt fraction (0.05–0.002 mm), and clay—clay fraction (< 0.002 mm in diameter). Multivariate PCA analysis for comparison of soil parameters and topographic factors between control and invaded plots. Table S2: Values of loadings and explained variance in PCA analysis of environmental variables. The loadings with highest value in particular PCA axis are bolded. Variable names abbreviation the same as in Table S2. Table S3: Results of statistical tests (Z, p, effect size) for functional traits between plots invaded by Lupinus polyphyllus and control plots for particular species (species). The significant differences are highlighted in bold. Additionally shown is the affinity of a species to plant functional types (plant type), number of observed pairs (N), as well as effect size. Table S4: Median values and changes (delta) in coordinates along the CSR triangle axes (strategy) for target species (species) in invaded and control plots with corresponding statistical test results (Z, p). Bolding letters indicate significant differences. Table S5: Spearman rank correlation matrix (r—upper triangle, p—lower triangle) among median of species height and effect size (ef) for height (ef‐height), leaf dry matter content (ef_LD [file ECE3-16-e73911-s001.zip › Fig.S7.JPG]

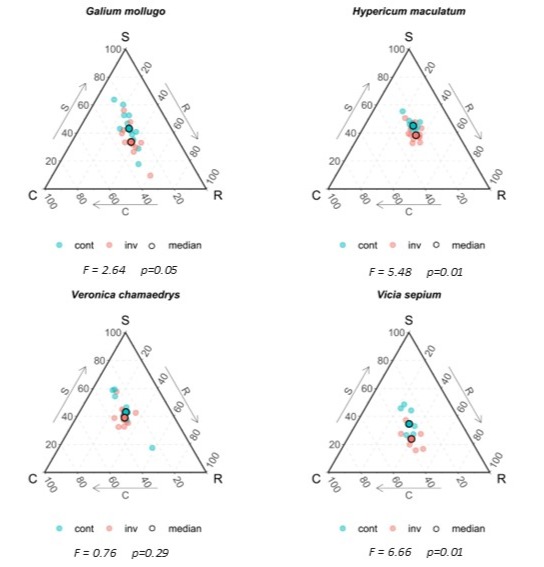

Supplement: Supplementary file 2 — Data S2: ece373911‐sup‐0002‐Supinfo.zip. Table S1: Geographical coordinates, spatial distance between paired plots at a site, species richness, aboveground biomass, and values of environmental variables for paired plots with results of statistical tests of differences between invaded and control plots (Chi2, p and distribution family). The abbreviations of variable names: N species—species richness, biomass—aboveground biomass, alt—altitude, TWI—topographic wetness index, DAH—diurnal anisotropic heating, N‐total nitrogen, C—total carbon, P—available phosphorus (P2O5), K—available potassium (K2O), Mg—available magnesium, pH—soil pH measured in a 1 M KCl solution, coarse—coarse soil fraction (particle diameter above 2 mm), sand—sand fraction (2–0.05 mm), silt—silt fraction (0.05–0.002 mm), and clay—clay fraction (< 0.002 mm in diameter). Multivariate PCA analysis for comparison of soil parameters and topographic factors between control and invaded plots. Table S2: Values of loadings and explained variance in PCA analysis of environmental variables. The loadings with highest value in particular PCA axis are bolded. Variable names abbreviation the same as in Table S2. Table S3: Results of statistical tests (Z, p, effect size) for functional traits between plots invaded by Lupinus polyphyllus and control plots for particular species (species). The significant differences are highlighted in bold. Additionally shown is the affinity of a species to plant functional types (plant type), number of observed pairs (N), as well as effect size. Table S4: Median values and changes (delta) in coordinates along the CSR triangle axes (strategy) for target species (species) in invaded and control plots with corresponding statistical test results (Z, p). Bolding letters indicate significant differences. Table S5: Spearman rank correlation matrix (r—upper triangle, p—lower triangle) among median of species height and effect size (ef) for height (ef‐height), leaf dry matter content (ef_LD [file ECE3-16-e73911-s001.zip › Fig.S8.JPG]

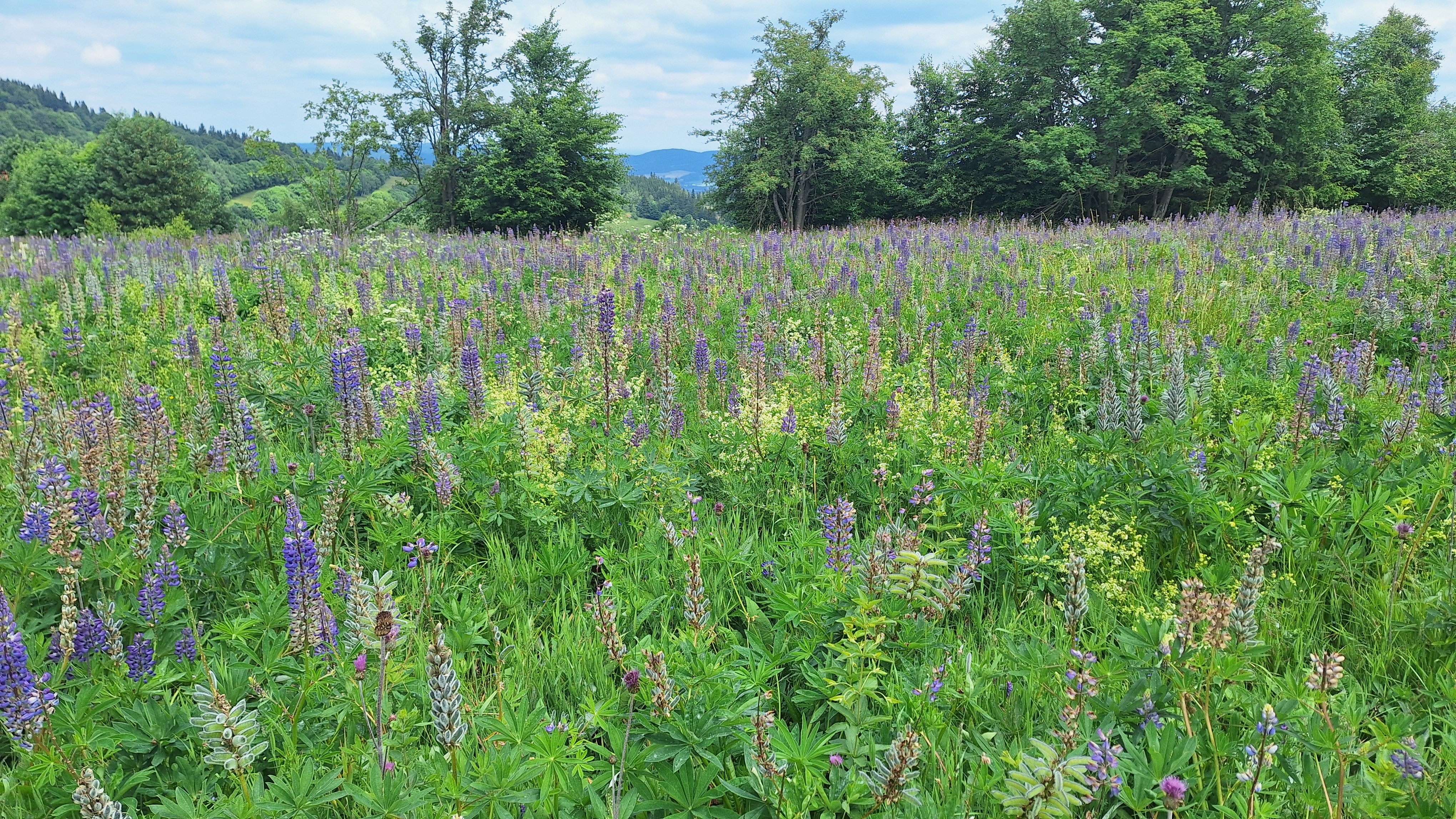

Supplement: Supplementary file 2 — Data S2: ece373911‐sup‐0002‐Supinfo.zip. Table S1: Geographical coordinates, spatial distance between paired plots at a site, species richness, aboveground biomass, and values of environmental variables for paired plots with results of statistical tests of differences between invaded and control plots (Chi2, p and distribution family). The abbreviations of variable names: N species—species richness, biomass—aboveground biomass, alt—altitude, TWI—topographic wetness index, DAH—diurnal anisotropic heating, N‐total nitrogen, C—total carbon, P—available phosphorus (P2O5), K—available potassium (K2O), Mg—available magnesium, pH—soil pH measured in a 1 M KCl solution, coarse—coarse soil fraction (particle diameter above 2 mm), sand—sand fraction (2–0.05 mm), silt—silt fraction (0.05–0.002 mm), and clay—clay fraction (< 0.002 mm in diameter). Multivariate PCA analysis for comparison of soil parameters and topographic factors between control and invaded plots. Table S2: Values of loadings and explained variance in PCA analysis of environmental variables. The loadings with highest value in particular PCA axis are bolded. Variable names abbreviation the same as in Table S2. Table S3: Results of statistical tests (Z, p, effect size) for functional traits between plots invaded by Lupinus polyphyllus and control plots for particular species (species). The significant differences are highlighted in bold. Additionally shown is the affinity of a species to plant functional types (plant type), number of observed pairs (N), as well as effect size. Table S4: Median values and changes (delta) in coordinates along the CSR triangle axes (strategy) for target species (species) in invaded and control plots with corresponding statistical test results (Z, p). Bolding letters indicate significant differences. Table S5: Spearman rank correlation matrix (r—upper triangle, p—lower triangle) among median of species height and effect size (ef) for height (ef‐height), leaf dry matter content (ef_LD [file ECE3-16-e73911-s001.zip › Fig_S9a.jpg]

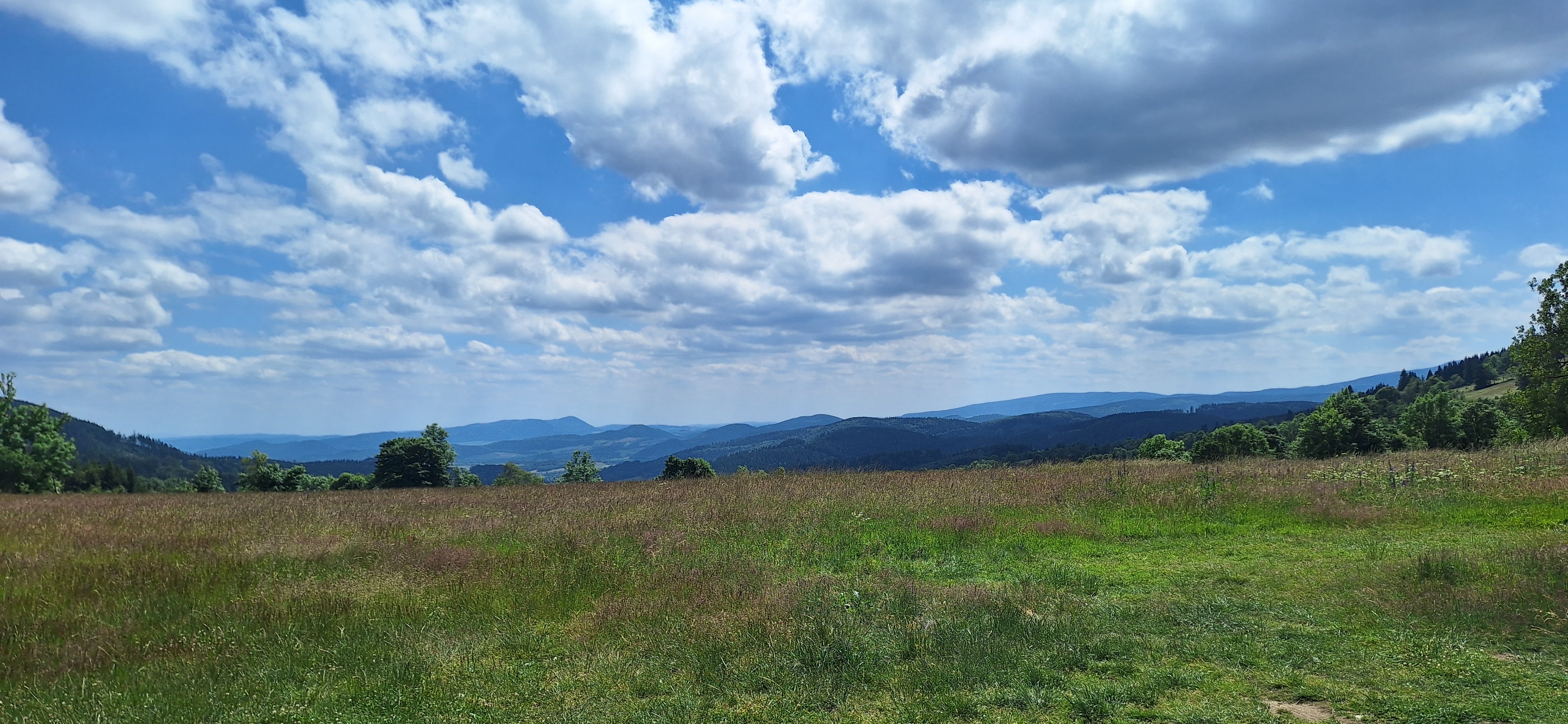

Supplement: Supplementary file 2 — Data S2: ece373911‐sup‐0002‐Supinfo.zip. Table S1: Geographical coordinates, spatial distance between paired plots at a site, species richness, aboveground biomass, and values of environmental variables for paired plots with results of statistical tests of differences between invaded and control plots (Chi2, p and distribution family). The abbreviations of variable names: N species—species richness, biomass—aboveground biomass, alt—altitude, TWI—topographic wetness index, DAH—diurnal anisotropic heating, N‐total nitrogen, C—total carbon, P—available phosphorus (P2O5), K—available potassium (K2O), Mg—available magnesium, pH—soil pH measured in a 1 M KCl solution, coarse—coarse soil fraction (particle diameter above 2 mm), sand—sand fraction (2–0.05 mm), silt—silt fraction (0.05–0.002 mm), and clay—clay fraction (< 0.002 mm in diameter). Multivariate PCA analysis for comparison of soil parameters and topographic factors between control and invaded plots. Table S2: Values of loadings and explained variance in PCA analysis of environmental variables. The loadings with highest value in particular PCA axis are bolded. Variable names abbreviation the same as in Table S2. Table S3: Results of statistical tests (Z, p, effect size) for functional traits between plots invaded by Lupinus polyphyllus and control plots for particular species (species). The significant differences are highlighted in bold. Additionally shown is the affinity of a species to plant functional types (plant type), number of observed pairs (N), as well as effect size. Table S4: Median values and changes (delta) in coordinates along the CSR triangle axes (strategy) for target species (species) in invaded and control plots with corresponding statistical test results (Z, p). Bolding letters indicate significant differences. Table S5: Spearman rank correlation matrix (r—upper triangle, p—lower triangle) among median of species height and effect size (ef) for height (ef‐height), leaf dry matter content (ef_LD [file ECE3-16-e73911-s001.zip › Fig_S9b.jpg]

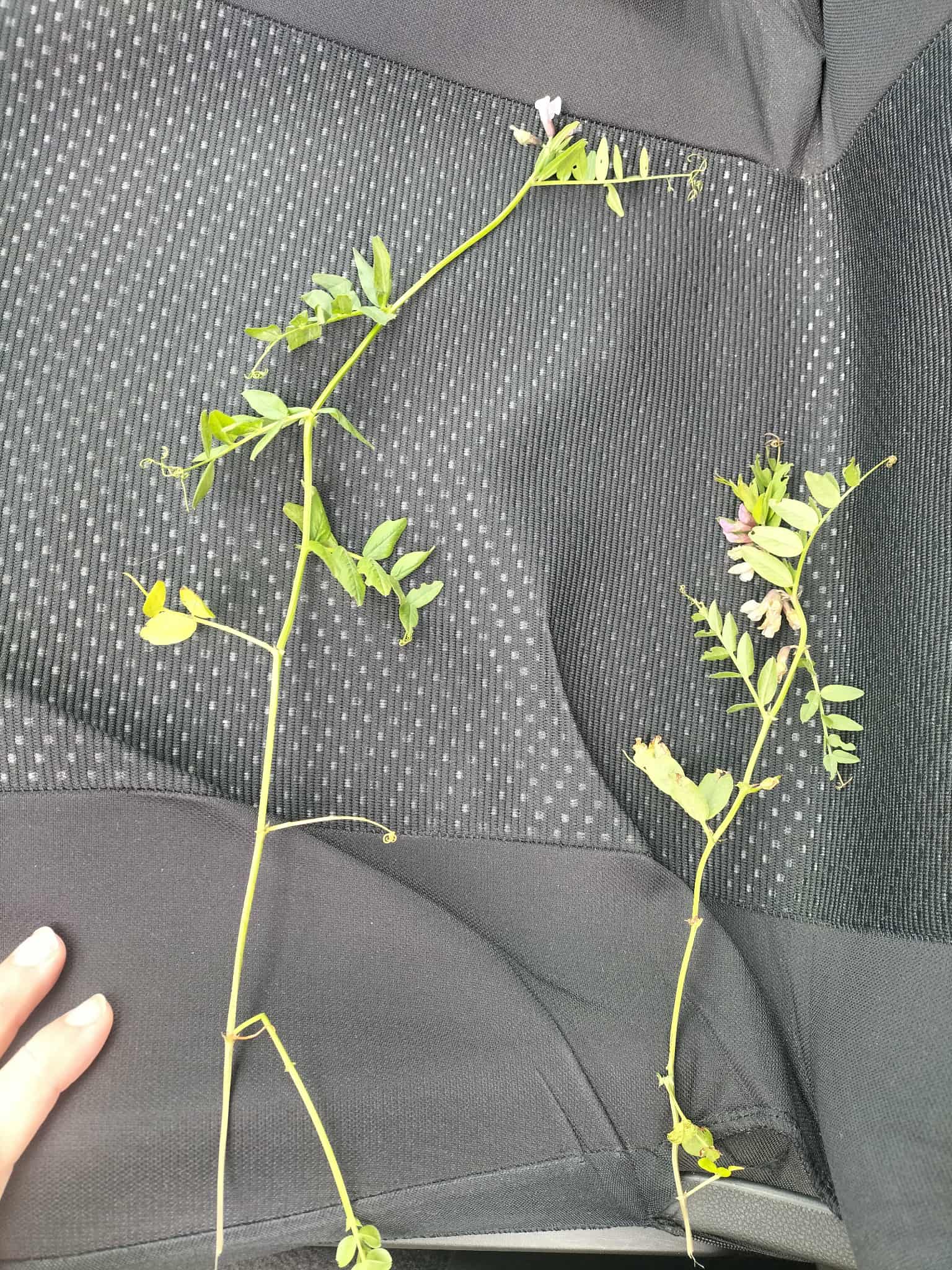

Supplement: Supplementary file 2 — Data S2: ece373911‐sup‐0002‐Supinfo.zip. Table S1: Geographical coordinates, spatial distance between paired plots at a site, species richness, aboveground biomass, and values of environmental variables for paired plots with results of statistical tests of differences between invaded and control plots (Chi2, p and distribution family). The abbreviations of variable names: N species—species richness, biomass—aboveground biomass, alt—altitude, TWI—topographic wetness index, DAH—diurnal anisotropic heating, N‐total nitrogen, C—total carbon, P—available phosphorus (P2O5), K—available potassium (K2O), Mg—available magnesium, pH—soil pH measured in a 1 M KCl solution, coarse—coarse soil fraction (particle diameter above 2 mm), sand—sand fraction (2–0.05 mm), silt—silt fraction (0.05–0.002 mm), and clay—clay fraction (< 0.002 mm in diameter). Multivariate PCA analysis for comparison of soil parameters and topographic factors between control and invaded plots. Table S2: Values of loadings and explained variance in PCA analysis of environmental variables. The loadings with highest value in particular PCA axis are bolded. Variable names abbreviation the same as in Table S2. Table S3: Results of statistical tests (Z, p, effect size) for functional traits between plots invaded by Lupinus polyphyllus and control plots for particular species (species). The significant differences are highlighted in bold. Additionally shown is the affinity of a species to plant functional types (plant type), number of observed pairs (N), as well as effect size. Table S4: Median values and changes (delta) in coordinates along the CSR triangle axes (strategy) for target species (species) in invaded and control plots with corresponding statistical test results (Z, p). Bolding letters indicate significant differences. Table S5: Spearman rank correlation matrix (r—upper triangle, p—lower triangle) among median of species height and effect size (ef) for height (ef‐height), leaf dry matter content (ef_LD [file ECE3-16-e73911-s001.zip › Fig_S10a.jpg]

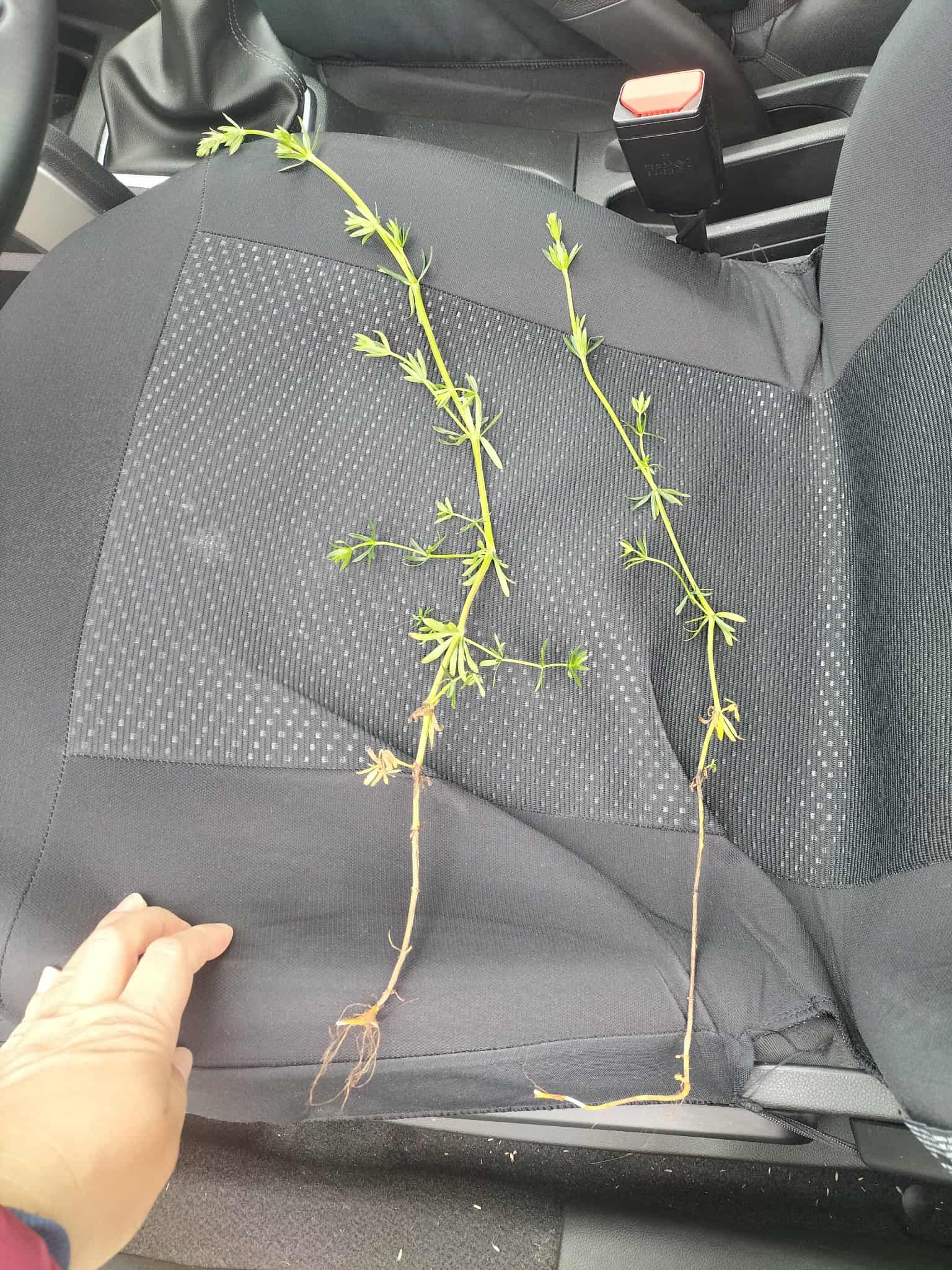

Supplement: Supplementary file 2 — Data S2: ece373911‐sup‐0002‐Supinfo.zip. Table S1: Geographical coordinates, spatial distance between paired plots at a site, species richness, aboveground biomass, and values of environmental variables for paired plots with results of statistical tests of differences between invaded and control plots (Chi2, p and distribution family). The abbreviations of variable names: N species—species richness, biomass—aboveground biomass, alt—altitude, TWI—topographic wetness index, DAH—diurnal anisotropic heating, N‐total nitrogen, C—total carbon, P—available phosphorus (P2O5), K—available potassium (K2O), Mg—available magnesium, pH—soil pH measured in a 1 M KCl solution, coarse—coarse soil fraction (particle diameter above 2 mm), sand—sand fraction (2–0.05 mm), silt—silt fraction (0.05–0.002 mm), and clay—clay fraction (< 0.002 mm in diameter). Multivariate PCA analysis for comparison of soil parameters and topographic factors between control and invaded plots. Table S2: Values of loadings and explained variance in PCA analysis of environmental variables. The loadings with highest value in particular PCA axis are bolded. Variable names abbreviation the same as in Table S2. Table S3: Results of statistical tests (Z, p, effect size) for functional traits between plots invaded by Lupinus polyphyllus and control plots for particular species (species). The significant differences are highlighted in bold. Additionally shown is the affinity of a species to plant functional types (plant type), number of observed pairs (N), as well as effect size. Table S4: Median values and changes (delta) in coordinates along the CSR triangle axes (strategy) for target species (species) in invaded and control plots with corresponding statistical test results (Z, p). Bolding letters indicate significant differences. Table S5: Spearman rank correlation matrix (r—upper triangle, p—lower triangle) among median of species height and effect size (ef) for height (ef‐height), leaf dry matter content (ef_LD [file ECE3-16-e73911-s001.zip › Fig_S10b.jpg]

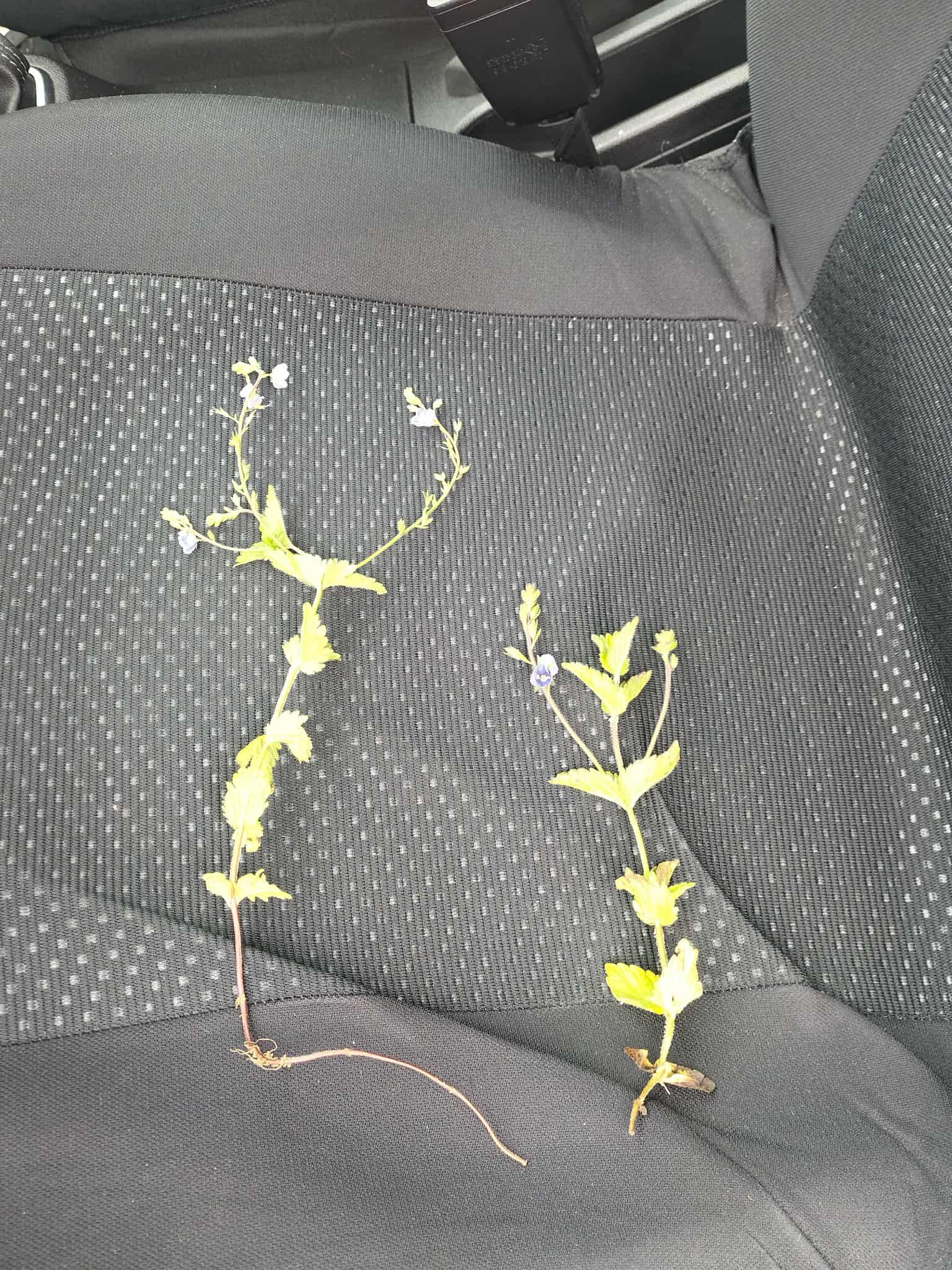

Supplement: Supplementary file 2 — Data S2: ece373911‐sup‐0002‐Supinfo.zip. Table S1: Geographical coordinates, spatial distance between paired plots at a site, species richness, aboveground biomass, and values of environmental variables for paired plots with results of statistical tests of differences between invaded and control plots (Chi2, p and distribution family). The abbreviations of variable names: N species—species richness, biomass—aboveground biomass, alt—altitude, TWI—topographic wetness index, DAH—diurnal anisotropic heating, N‐total nitrogen, C—total carbon, P—available phosphorus (P2O5), K—available potassium (K2O), Mg—available magnesium, pH—soil pH measured in a 1 M KCl solution, coarse—coarse soil fraction (particle diameter above 2 mm), sand—sand fraction (2–0.05 mm), silt—silt fraction (0.05–0.002 mm), and clay—clay fraction (< 0.002 mm in diameter). Multivariate PCA analysis for comparison of soil parameters and topographic factors between control and invaded plots. Table S2: Values of loadings and explained variance in PCA analysis of environmental variables. The loadings with highest value in particular PCA axis are bolded. Variable names abbreviation the same as in Table S2. Table S3: Results of statistical tests (Z, p, effect size) for functional traits between plots invaded by Lupinus polyphyllus and control plots for particular species (species). The significant differences are highlighted in bold. Additionally shown is the affinity of a species to plant functional types (plant type), number of observed pairs (N), as well as effect size. Table S4: Median values and changes (delta) in coordinates along the CSR triangle axes (strategy) for target species (species) in invaded and control plots with corresponding statistical test results (Z, p). Bolding letters indicate significant differences. Table S5: Spearman rank correlation matrix (r—upper triangle, p—lower triangle) among median of species height and effect size (ef) for height (ef‐height), leaf dry matter content (ef_LD [file ECE3-16-e73911-s001.zip › Fig_S10c.jpg]

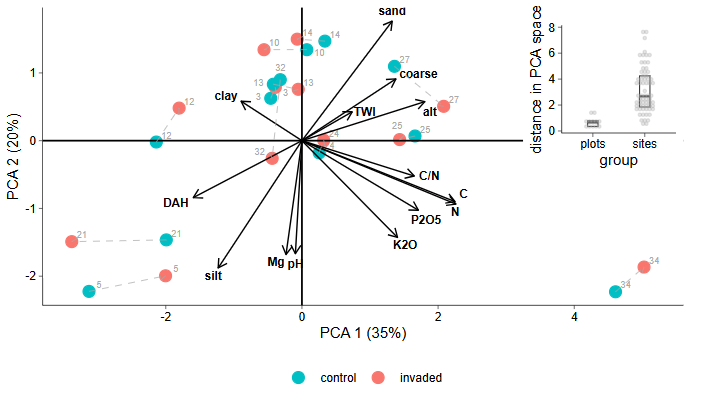

Supplement: Supplementary file 2 — Data S2: ece373911‐sup‐0002‐Supinfo.zip. Table S1: Geographical coordinates, spatial distance between paired plots at a site, species richness, aboveground biomass, and values of environmental variables for paired plots with results of statistical tests of differences between invaded and control plots (Chi2, p and distribution family). The abbreviations of variable names: N species—species richness, biomass—aboveground biomass, alt—altitude, TWI—topographic wetness index, DAH—diurnal anisotropic heating, N‐total nitrogen, C—total carbon, P—available phosphorus (P2O5), K—available potassium (K2O), Mg—available magnesium, pH—soil pH measured in a 1 M KCl solution, coarse—coarse soil fraction (particle diameter above 2 mm), sand—sand fraction (2–0.05 mm), silt—silt fraction (0.05–0.002 mm), and clay—clay fraction (< 0.002 mm in diameter). Multivariate PCA analysis for comparison of soil parameters and topographic factors between control and invaded plots. Table S2: Values of loadings and explained variance in PCA analysis of environmental variables. The loadings with highest value in particular PCA axis are bolded. Variable names abbreviation the same as in Table S2. Table S3: Results of statistical tests (Z, p, effect size) for functional traits between plots invaded by Lupinus polyphyllus and control plots for particular species (species). The significant differences are highlighted in bold. Additionally shown is the affinity of a species to plant functional types (plant type), number of observed pairs (N), as well as effect size. Table S4: Median values and changes (delta) in coordinates along the CSR triangle axes (strategy) for target species (species) in invaded and control plots with corresponding statistical test results (Z, p). Bolding letters indicate significant differences. Table S5: Spearman rank correlation matrix (r—upper triangle, p—lower triangle) among median of species height and effect size (ef) for height (ef‐height), leaf dry matter content (ef_LD [file ECE3-16-e73911-s001.zip › FigS1.tiff]
